# ML290 is a biased allosteric agonist at the relaxin receptor RXFP1

**DOI:** 10.1038/s41598-017-02916-5

**Published:** 2017-06-07

**Authors:** Martina Kocan, Mohsin Sarwar, Sheng Y. Ang, Jingbo Xiao, Juan J. Marugan, Mohammed A. Hossain, Chao Wang, Dana S. Hutchinson, Chrishan S. Samuel, Alexander I. Agoulnik, Ross A. D. Bathgate, Roger J. Summers

**Affiliations:** 1 Drug Discovery Biology, Monash Institute of Pharmaceutical Sciences, Monash University, Parkville, Australia; 2 Preclinical Innovation, National Center for Advancing Translational Sciences, National Institutes of Health, Maryland, USA; 3 The Florey Institute of Neuroscience and Mental Health, Parkville, Australia; 4 Department of Biochemistry and Molecular Biology, University of Melbourne, Parkville, Australia; 5 Cardiovascular Disease Program, Biomedicine Discovery Institute and Department of Pharmacology, Monash University, Clayton, Australia; 6 Department of Human and Molecular Genetics, Herbert Wertheim College of Medicine, Florida International University, Florida, USA

## Abstract

Activation of the relaxin receptor RXFP1 has been associated with improved survival in acute heart failure. ML290 is a small molecule RXFP1 agonist with simple structure, long half-life and high stability. Here we demonstrate that ML290 is a biased agonist in human cells expressing RXFP1 with long-term beneficial actions on markers of fibrosis in human cardiac fibroblasts (HCFs). ML290 did not directly compete with orthosteric relaxin binding and did not affect binding kinetics, but did increase binding to RXFP1. In HEK-RXFP1 cells, ML290 stimulated cAMP accumulation and p38MAPK phosphorylation but not cGMP accumulation or ERK1/2 phosphorylation although prior addition of ML290 increased p-ERK1/2 responses to relaxin. In human primary vascular endothelial and smooth muscle cells that endogenously express RXFP1, ML290 increased both cAMP and cGMP accumulation but not p-ERK1/2. In HCFs, ML290 increased cGMP accumulation but did not affect p-ERK1/2 and given chronically activated MMP-2 expression and inhibited TGF-β1-induced Smad2 and Smad3 phosphorylation. In vascular cells, ML290 was 10x more potent for cGMP accumulation and p-p38MAPK than for cAMP accumulation. ML290 caused strong coupling of RXFP1 to Gα_s_ and Gα_oB_ but weak coupling to Gα_i3_. ML290 exhibited signalling bias at RXFP1 possessing a signalling profile indicative of vasodilator and anti-fibrotic properties.

## Introduction

In a recently completed phase III clinical trial (RELAX-AHF), serelaxin a recombinant form of the major stored and circulating form of human relaxin 2 (H2) gene, reduced overall mortality and provided rapid relief of congestion as well as reducing organ damage^1, 2^. These effects likely reflect the cardioprotective actions of H2 relaxin that include vasodilation, angiogenesis, anti-inflammatory and anti-fibrotic effects that have been shown in experimental models of cardiovascular disease^3^.

One likely target of H2 relaxin in humans is the vasculature because H2 relaxin has potent vasodilatory and anti-fibrotic effects in human and rodent isolated blood vessels^4, 5^. At the cellular level, H2 relaxin binds to orthosteric binding sites in the leucine rich repeat (LRR) region and extracellular loop 2 (ECL2) leading to signal transduction in human umbilical vascular cells where it acutely activates cAMP, cGMP and p-ERK1/2 signalling and in the longer-term, increases the expression of nNOS, ET_B_ and VEGFA^6^. In addition, H2 relaxin abrogates fibrosis and prevents and/or reverses aberrant collagen deposition in numerous experimental models of disease, regardless of etiology^7–9^. Despite the clinical promise of H2 relaxin, it has limitations as a therapeutic including cross-reactivity with other relaxin family peptide receptors^9^, no oral bioavailability and a short half-life of <10 min^10^, requiring long term i.v. or s.c. infusions to produce a therapeutic effect. Therefore the development of selective and orally bioavailable agonists of RXFP1 has potential significant benefits.

ML290 is the first small molecule agonist selective for RXFP1^11–13^. It increases cAMP accumulation and VEGF expression in cells that endogenously express human RXFP1 but not in cells that express RXFP2 or RXFP3^13^. In contrast to H2 relaxin, ML290 has a plasma half-life of 8.56 hr in mice without obvious toxicity^13^. ML290 activates human, monkey and pig RXFP1, with no agonist actions at the mouse orthologue^11^, failing to compete directly for orthosteric ^125^I-H2 relaxin binding to human RXFP1, suggesting an allosteric site of action^13^. Recent studies demonstrate that the binding site of ML290 is located in a binding pocket formed by the TM domains displaying a strong hydrophobic interaction at the extracellular end of TM7 and forming a particularly important hydrogen bond interaction with the ECL3 residues G659/T660^11^. To date, there is no detailed information available on the signal transduction mechanisms utilised by ML290 in recombinant cell lines or in cells that endogenously express RXFP1.

With this in mind, we have examined the binding and signalling profiles of ML290 in comparison with H2 relaxin. We measured cAMP accumulation, cGMP accumulation, p-ERK1/2 and p38MAPK phosphorylation (p-p38MAPK) in HEK293T cells stably expressing RXFP1 (HEK-RXFP1) and in human primary vascular cells. Moreover, we also investigated the potential anti-fibrotic properties of ML290 by evaluating its ability to promote markers such as matrix metalloproteinase (MMP)-2 and inhibit the pro-fibrotic actions of TGF-β1-induced Smad-2 and Smad-3 phosphorylation in primary human cardiac fibroblasts, representing key fibrosis-producing cells.

## Results

### Alteration of the binding characteristics of ^125^I-H2 relaxin by ML290 confirms an allosteric interaction with RXFP1

**Figure 1:**
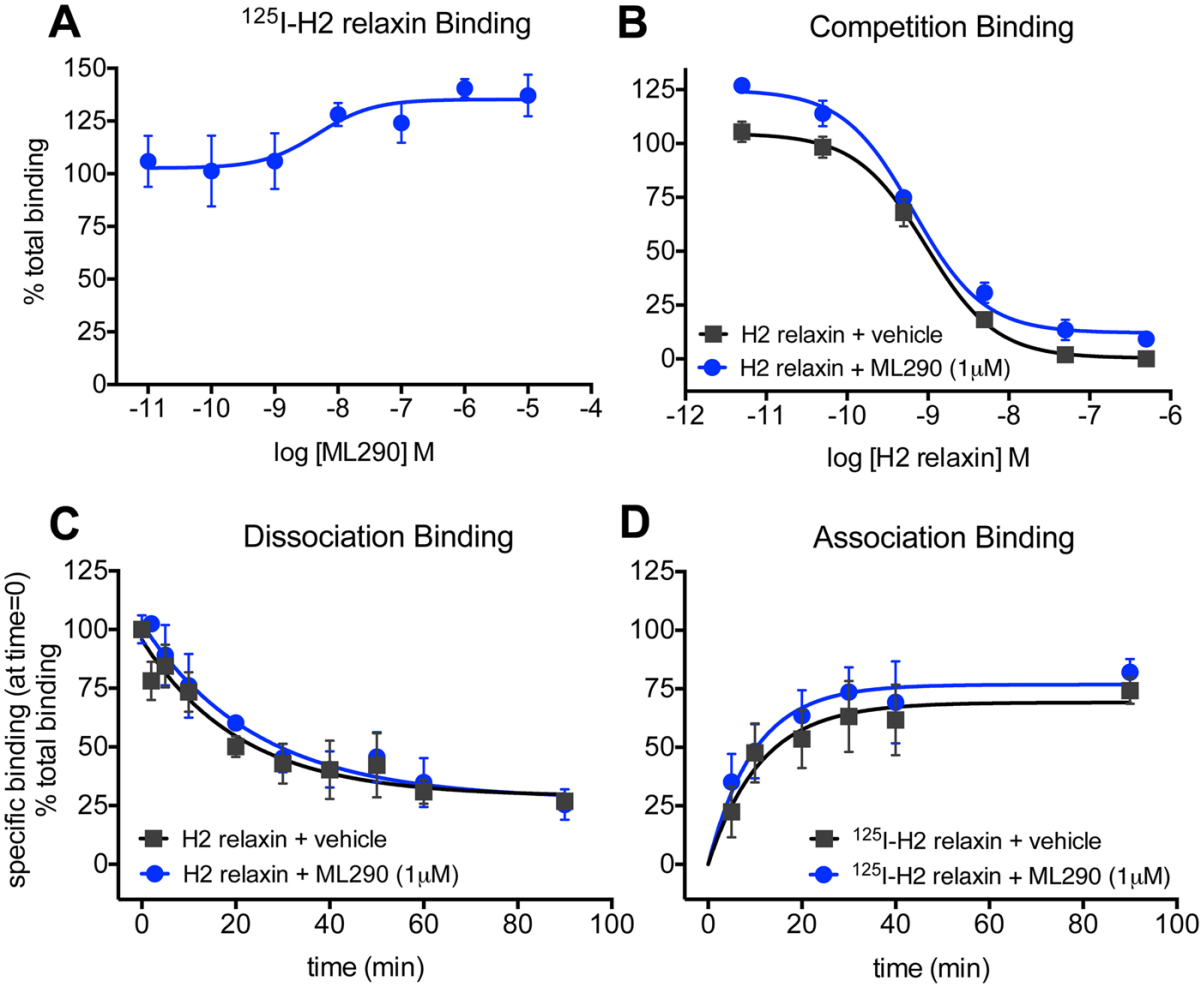
ML290 and binding of ^125^I-H2 relaxin to RXFP1 stably expressed in HEK293 cells. In (**A**) co-incubation with ML290 (90 min), increased total binding of ^125^I-H2 relaxin to RXFP1 in a concentration-dependent manner (n = 6). In (**B**) ML290 (1 μM) had no significant effect on competition by H2 relaxin for ^125^I-H2 relaxin binding (n = 8). In kinetic studies, ML290 (1 μM) had no significant effect on (**C**) dissociation (n = 3) or (**D**) association rate (n = 6–7) of ^125^I-H2 relaxin from RXFP1. Data are mean ± SEM of ‘n’ experiments.

ML290 does not compete for ^125^I-H2 relaxin binding^13^ at the human RXFP1 and since there is strong evidence from mutation studies that it binds to a topographically distinct site from that of H2 relaxin and displays species specificity^14^, it suggests an allosteric mode of action. Examination of the binding profile in more detail in HEK-RXFP1 cells incubated with ^125^I-H2 relaxin (100pM), showed that ML290 concentration-dependently increased specific binding (pEC_50_: 8.8 ± 0.7) to RXFP1 from 102.7 ± 7.3% to 135.2 ± 6.5% (p = 0.04) (Fig. 1A). In competition studies, ML290 (1 μM) had no significant effect on the affinity of H2 relaxin (H2 relaxin pK_i_ 9.5 ± 0.1 cf H2 relaxin + ML290 pK_i_ 9.7 ± 0.1, Fig. 1B) confirming previous studies^13^. In kinetic studies, ML290 (1 μM) had no significant effect on the dissociation rate of ^125^I-H2 relaxin in HEK-RXFP1 cells (H2 relaxin K_off_ 0.05 ± 0.02 min^−1^ cf H2 relaxin + ML290 K_off_ 0.04 ± 0.01 min^−1^, Fig. 1C), nor on the association rate (H2 relaxin K_on_ 2.1 × 10^8^ M^−1^ min^−1^ cf H2 relaxin + ML290 K_on_ 2.8 × 10^8^ M^−1^ min^−1^; Fig. 1D).

### Comparison of the G-protein coupling profile produced H2 relaxin and ML290

There is evidence using pertussis toxin resistant mutants of G proteins or specific G protein inhibitors, that H2 relaxin promotes coupling of RXFP1 to Gα_s_, Gα_i3_ and Gα_o/B_ in HEK-RXFP1 cells and in human primary vascular cells^6, 15^. To determine the G-proteins involved in ML290-mediated signal transduction we have used real-time kinetic BRET in HEK RXFP1 cells to directly examine ligand-induced interactions between RXFP1 and G-proteins.

**Figure 2:**
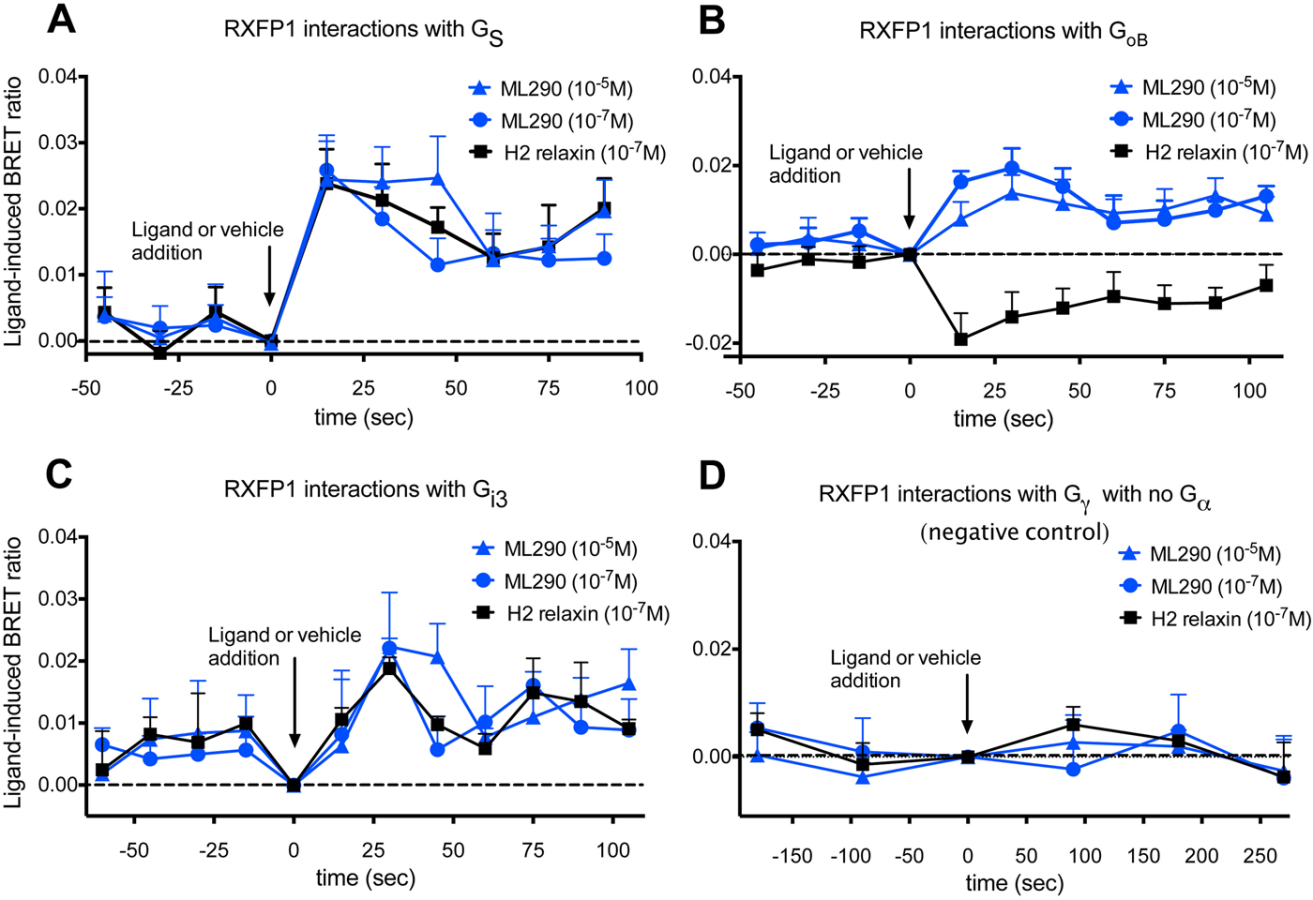
RXFP1 - G protein interactions and treatment with H2 relaxin or ML290. HEK-RXFP1-Rluc8 cells were transiently co-transfected with Gγ2-Venus, Gβ1 and one of Gα subunits (Gα_s_, Gα_oB_, Gα_i3_). Interactions between RXFP1 and G proteins were detected prior to and after treatment with H2 relaxin (0.1 μM) or ML290 (0.1 μM or 10 μM) using real-time BRET assays. Both ML290 and H2 relaxin induced interactions between RXFP1-Rluc8 and Gα_s_, Gα_oB_, and to a lesser extent Gα_i3_ (**A**–**C**). Shifts in BRET ratio between RXFP1 and Gα_s_ were quantitatively and qualitatively similar with ML290 and H2 relaxin. However the ML290 RXFP1- Gα_oB_ ligand-induced BRET ratio moved in the opposite direction to that to H2 relaxin (**B**) suggesting that each ligand induces a different receptor conformation. Interactions between RXFP1 and Gα_i3_ with H2 relaxin and ML290 were weak but qualitatively similar. There were no interactions between RXFP1-Rluc8 and Gγ2-Venus in the absence of Gα subunits (**D**). Ligand-induced BRET ratios were calculated by subtracting the BRET ratio for the vehicle-treated sample from that obtained from each ligand-treated sample as described in Materials and Methods. Data are mean ± SEM of 4 independent experiments.

HEK293T cells co-expressing RXFP1-Rluc8, G_γ2_-Venus, G_β1_ and one of three Gα subunits (Gα_s_, Gα_oB_, Gα_i3_, or no Gα) were examined after treatment with H2 relaxin (100 nM) or ML290 (100 nM or 10 μM). H2 relaxin and ML290 promoted interactions between RXFP1 and Gα_s_, Gα_o/B_ and to a lesser extent Gα_i3_ (Fig. 2). The profile of ML290 and H2 relaxin-induced BRET between RXFP1 and Gα_s_ was almost identical (Fig. 2A) whereas the profile for ML290-induced BRET for RXFP1-Gα_oB_ interactions clearly differed from that observed for H2 relaxin (Fig. 2B). Both ligands produced a small but similar response with Gα_i3_ (Fig. 2C). These interactions were specific for RXFP1 and Gαs, Gα_oB_ and Gα_i3_ because control studies in HEK293T cells co-expressing RXFP1-Rluc8, G_γ2_-Venus, G_β1_ but no Gα subunits, showed no H2 relaxin or ML290-induced BRET (Fig. 2D). All G-protein constructs used in this study have been validated and shown to be functional^16^.

### MAPK and cAMP activation by H2 relaxin and ML290 in HEK-RXFP1 cells

Time course studies showed that ERK1/2 phosphorylation (p-ERK1/2) was observed with H2 relaxin (100 nM) but not with direct addition of ML290 (100 nM or 10 μM) (Figure S1A). Both H2 relaxin (100 nM) and ML290 (100 nM or 10 μM) increased p38MAPK phosphorylation (Figure S1B) but had no effect on JNK1/2 phosphorylation (p-JNK1/2/3) (Figure S1C). These responses were mediated by RXFP1 as all ligands failed to cause p-ERK1/2 and p-p38MAPK responses in HEK293T cells lacking RXFP1 (data not shown).

**Figure 3:**
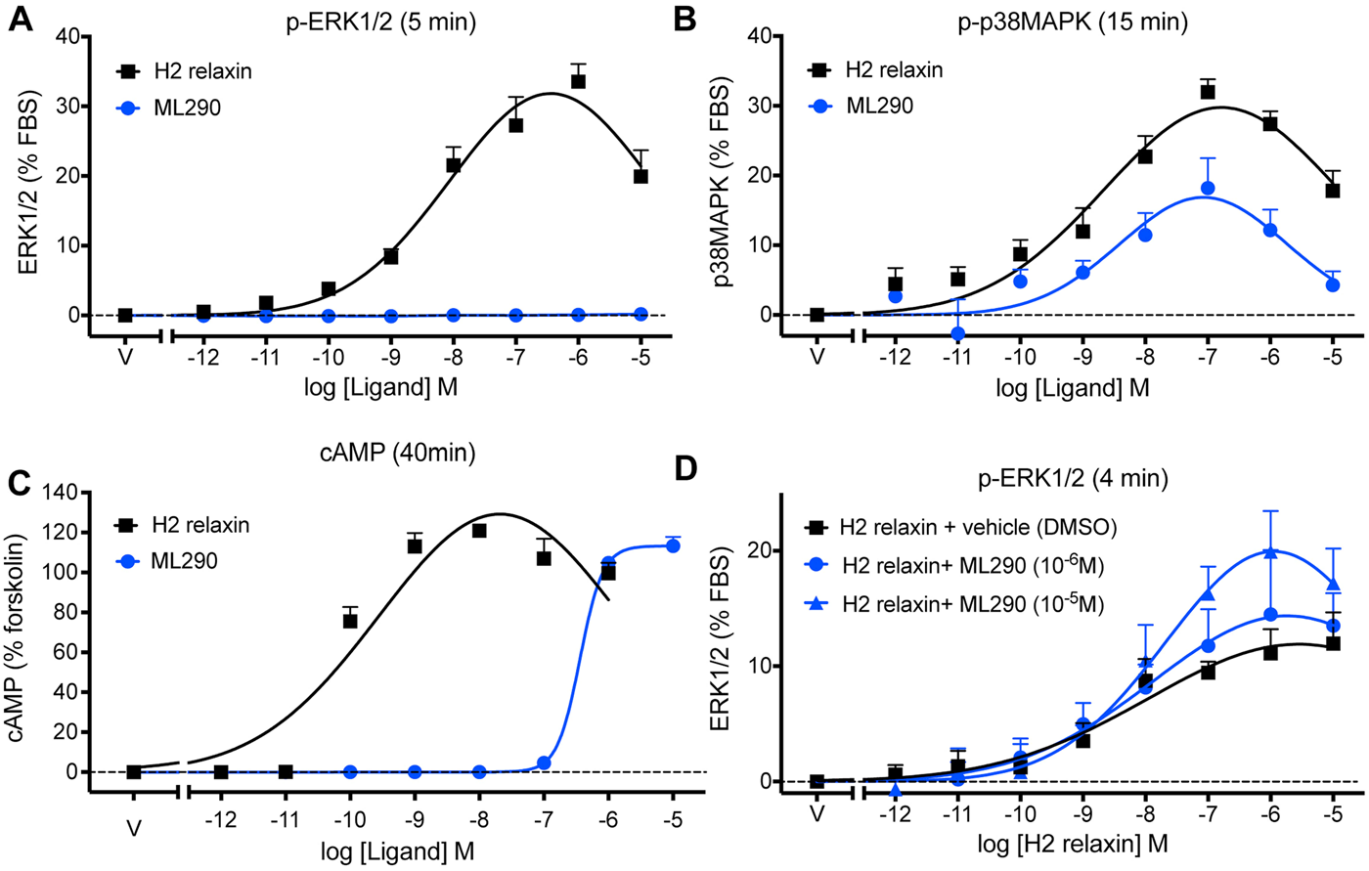
Activation of ERK1/2, p38MAPK and generation of cAMP by H2 relaxin and ML290. In HEK-RXFP1 cells, H2 relaxin activated p-ERK1/2 (**A**) 5 min), p-p38MAPK (**B**) 15 min) and cAMP accumulation (**C**) 30 min) in a concentration-dependent manner. ML290 did not directly activate p-ERK1/2 (**A**), but did activate p38MAPK (**B**) with lower efficacy and cAMP accumulation with similar efficacy but significantly lower potency than H2 relaxin (**C**). 10 min pretreatment with ML290 enhanced p-ERK1/2 activation produced by relaxin (**D**) 4 min). Data are mean ± SEM for 4–8 independent experiments.

Concentration-response relationships for p-ERK1/2 at 5 min showed that H2 relaxin had a high potency (pEC_50_: 9.5 ± 0.3; Table 1) and efficacy (E_max_: 31.8 ± 1.7% of the response to 10% FBS) whereas ML290 failed to produce a response (Fig. 3A). However, both H2 relaxin (pEC_50_: 8.9 ± 0.3) and ML290 (pEC_50_: 9.3 ± 0.6) produced p-p38MAPK responses at 15 min with similar potency (NS, Table 1) although H2 relaxin had higher efficacy (E_max_ H2 relaxin: 29.8 ± 1.7% cf E_max_ ML290: 16.9 ± 2.2%; P < 0.005) (Fig. 3B). ML290 was a weaker agonist for cAMP accumulation in HEK-RXFP1 cells than H2 relaxin. Incubation with ML290 or H2 relaxin for 30 min increased cAMP accumulation in HEK-RXFP1 cells to a maximum of 113.3 ± 1.8% and 129.0 ± 5.8% of the response to forskolin (50 μM), respectively (Fig. 3C). H2 relaxin had significantly higher potency (pEC_50_: 10.3 ± 0.1) than ML290 (pEC_50_: 6.4 ± 0.1; P < 0.0001; Table 1) despite having similar efficacy (Fig. 3C). Pretreatment (10 min) with ML290 (10 μM) significantly increased the maximum p-ERK1/2 response to relaxin without altering potency (Fig. 3D).

**Table 1:** The potency of ML290 and relaxin in HEK-RXFP1 and human primary vascular cells for p-ERK1/2, pp38MAPK, cGMP and cAMP accumulation.

	p-ERK1/2 (% 10% FBS)		p-p38MAPK (% sorbitol)	
	H2 relaxin	ML290	H2 relaxin	ML290
HEK-RXFP1	9.5 ± 0.3 (4) (31.8 ± 1.7)	NE (8)	8.9 ± 0.3 (4) (29.8 ± 1.7)	9.3 ± 0.6 (6) (16.9 ± 2.2)
HCAEC	ND	NE (3)	ND	NE (3)
HUVEC	9.2 ± 0.3^#^ (5) (11.2 ± 2.7)	NE (3)	ND	NE (3)
HUASMC	9.1 ± 0.4^#^ (6) (27.7 ± 8.1)	NE (3)	ND	8.6 ± 0.6 (3) (4.4 ± 1.6)
HUVSMC	9.2 ± 0.4^#^ (6) (15.2 ± 4.4)	NE (3)	ND	8.5 ± 1.0 (3) (2.7 ± 0.9)
HCF	9.1 ± 0.3^#^ (5) (54.7 ± 15.9)	NE (3)	ND	ND
	**cGMP (% DEA 1 μM)**		**cAMP (% forskolin 50 μM)**	
HEK-RXFP1	NE (3)	NE (3)	10.3 ± 0.1 (5) (129.0 ± 5.8)	6.4 ± 0.1 (6) (113.3 ± 1.8)
HCAEC	9.2 ± 0.5 (3) (39.6 ± 14.8)	7.2 ± 0.4 (7) (40.6 ± 5.6)	8.9 ± 0.5 (3) (13.0 ± 5.2)	6.1 ± 0.3 (7) (17.9 ± 3.3)
HUVEC	8.9 ± 0.4^#^ (7) (65.4 ± 19.8)	7.3 ± 0.5 (7) (23.5 ± 4.4)	9.1 ± 0.4^#^ (9) (6.0 ± 2.7)	6.2 ± 0.7 (7) (11.7 ± 3.9)
HUASMC	9.1 ± 0.3^#^ (6) (55.7 ± 12.4)	7.2 ± 0.5 (7) (21.0 ± 4.1)	9.0 ± 0.3^#^ (7) (4.5 ± 1.6)	6.2 ± 0.5 (5) (11.9 ± 3.9)
HUVSMC	9.2 ± 0.4^#^ (6) (31.4 ± 10.9)	7.2 ± 0.6 (4) (11.0 ± 3.4)	9.6 ±0.4^#^ (10) (5.1 ± 1.8)	6.1 ± 0.5 (4) (10.8 ± 3.0)
HCF	9.6 ± 0.7 (5) (62.3 ± 7.6)	7.5± 0.3 (5) (45.3 ± 4.1)	NE (3)	ND

Note: ^#^Indicates data taken from our previous publication conducted in the same cells^6^. Efficacy (below in brackets) expressed as a % of control responses for each pathway. N numbers in brackets. NE – no effect; ND – not determined.

### In human primary cells, ML290 causes cAMP and cGMP accumulation but not ERK1/2 phosphorylation in smooth muscle and endothelial cells and cGMP accumulation but not ERK1/2 phosphorylation in cardiac fibroblasts

**Figure 4:**
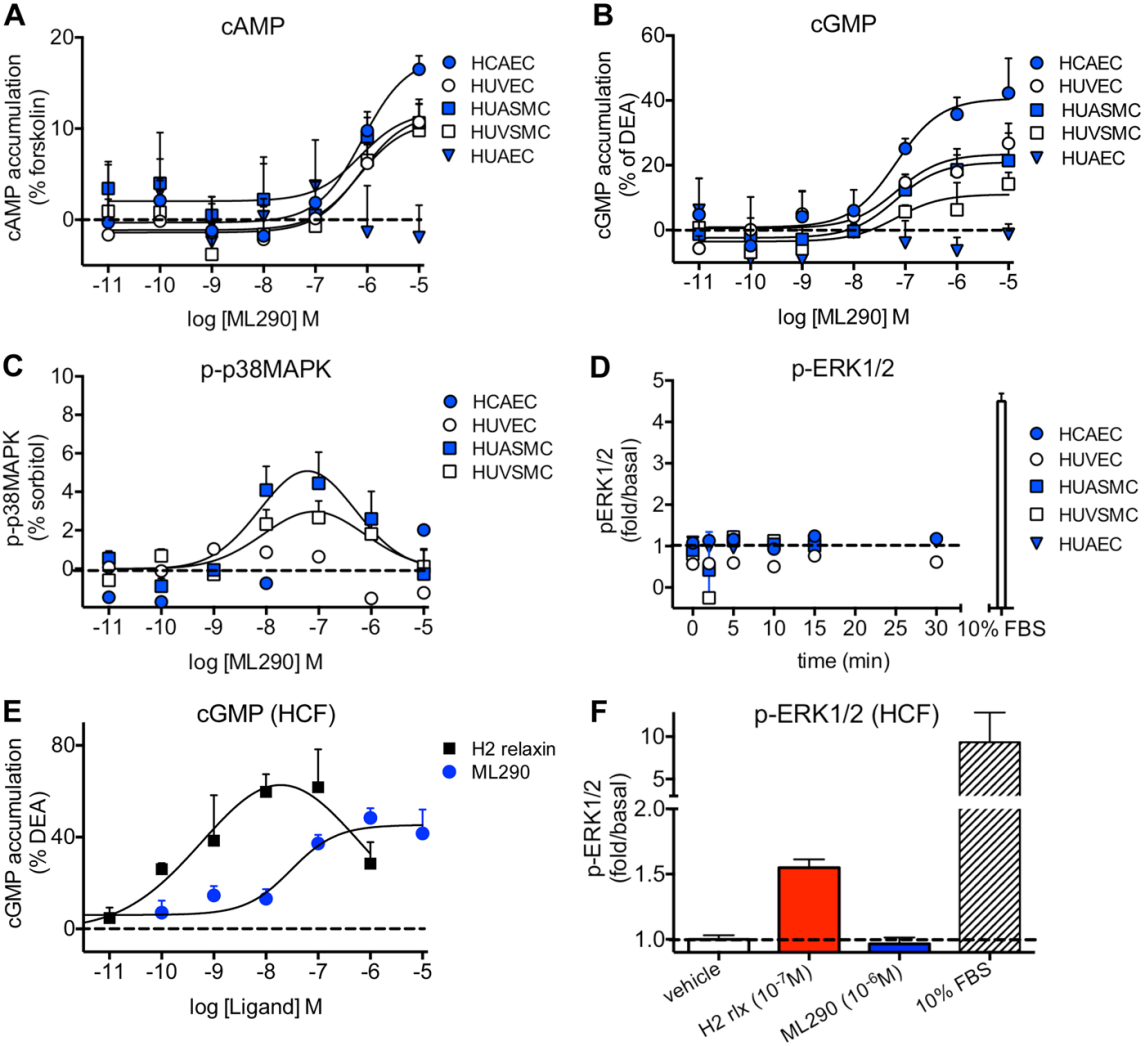
Signal transduction pathways activated by ML290 in human primary vascular cells and in human cardiac fibroblasts (HCF). In (**A**) ML290 (30 min) concentration-dependently increased cAMP accumulation in HCAECs (, n = 7), HUVECs (, n = 7), HUASMCs (, n = 5), HUVSMCs (, n = 4) but not in HUAECs that do not express cell surface RXFP1 (, n = 4); in (**B**) ML290 (30 min) also increased cGMP accumulation in HCAECs (n = 7), HUVECs (n = 7), HUASMCs (n = 7), HUVSMCs (n = 4) but not in HUAECs (n = 4); In (**C**) ML290 increased p-p38MAPK (15 min) but only in HUASMC and HUVSMCs (n = 3); In (**D**) ML290 did not cause ERK1/2 phosphorylation in HCAECs (n = 3), HUVECs (n = 3), HUASMCs (n = 3), HUVSMCs (n = 3) or HUAECs (n = 3). In HCFs, H2 relaxin activated cGMP accumulation in a concentration-dependent manner ((**E**) n = 5) (40 min) and p-ERK1/2 ((**F**) n = 3) (5 min). ML290 did not activate p-ERK1/2 (F; n = 3) but did induce cGMP accumulation (E; n = 5) in a concentration-dependent manner albeit with lower potency than H2 relaxin. Data shown are mean ± SEM of ‘n’ independent experiments.

H2 relaxin is known to increase cAMP accumulation in HUVEC, HUASMC and HUVSMC^6, 17^ (Table 1). ML290 (30 min) also concentration-dependently increased cAMP accumulation (Fig. 4A) up to 17.9 ± 3.3% of the forskolin response (50 μM, 30 min) in HCAECs (pEC_50_: 6.1 ± 0.3), 11.7 ± 3.9% in HUVECs (pEC_50_: 6.2 ± 0.7), 10.8 ± 3.0% in HUVSMCs (pEC_50_: 6.1 ± 0.5) and 11.9 ± 3.9% in HUASMCs (pEC_50_: 6.2 ± 0.5). ML290 was 3 orders of magnitude less potent than H2 relaxin (Table 1). ML290 did not cause cAMP accumulation in HUAEC (Fig. 4A), cells known not to express cell surface RXFP1^6^.

Although the concentration-response curve (CRC) to H2 relaxin in HEK-RXFP1 cells was bell-shaped (Fig. 3C) as in human primary vascular cells^6^, that to ML290 was right-shifted and incomplete due to limitations on the concentration that could be added, and therefore no conclusion can be reached regarding the shape of the ML290 concentration-response curve.

H2 relaxin also activates cGMP signalling in a variety of cells^18, 19^ including human primary vascular cells^6^. ML290 (30 min) also concentration-dependently increased cGMP accumulation (Fig. 4B) to 40.6 ± 5.6% of the DEA response (10 μM, 5 min) in HCAECs (pEC_50_: 7.2 ± 0.4), 23.5 ± 4.4% in HUVECs (pEC_50_: 7.3 ± 0.5), 11.0 ± 3.4% in HUVSMCs (pEC_50_: 7.2 ± 0.6) and 21.0 ± 4.1% in HUASMCs (pEC_50_: 7.2 ± 0.5). ML290 had no effect on cGMP accumulation in HUAEC (Fig. 4B), the cells that do not express cell surface RXFP1^6^. It was of interest to note that the potency of ML290 in the cGMP assay was greater than an order of magnitude higher than that observed in the cAMP assay (Table 1). In order to determine whether the vehicle for ML290 affected the potency of ligands acting at RXFP1, HCAECs, HUVECs, HUASMCs and HUVSMCs were treated with H2 relaxin and DMSO (1%) which had no significant effect on potency or efficacy for cAMP or cGMP accumulation (Figure S2).

ML290 concentration-dependently increased p-p38MAPK (Fig. 4C) in HUASMC to 4.4 ± 1.6% of the sorbitol response (pEC_50_: 8.6 ± 0.6) and to 2.7 ± 0.9% of the sorbitol response in HUVSMC (pEC_50_: 8.5 ± 1.0) (Table 1) but did not activate p-p38MAPK in HUVEC and HCAEC despite ML290 causing a cAMP (Fig. 4A) and cGMP (Fig. 4B) response in these cells.

H2 relaxin is known to stimulate a cGMP responses in HCFs^6^. Here, we have shown that ML290 also produces concentration-dependent cGMP accumulation in HCFs (Fig. 4E). ML290 induced cGMP signalling with somewhat lower efficacy and potency (E_max_: 45.3 ± 4.1% of the response to 10  μM DEA and pEC_50_: 7.5 ± 0.3) compared to H2 relaxin (E_max_: 62.3 ± 7.6% of the response to 10  μM DEA and pEC_50_: 9.6 ± 0.7).

H2 relaxin increases p-ERK1/2 in human primary vascular cells and in HCFs (confirmed here, Fig. 4F)^6^. However, in addition to HEK-RXFP1 cells, ML290 had no effect when added directly on p-ERK1/2 in HCAECs, HUVECs, HUASMCs and HUVSMCs (Fig. 4D) or in HCF whereas the positive control (FBS: 10%, 5 min) caused a 4-fold increase in human primary vascular cells (Fig. 4D) and a 9-fold increase in p-ERK1/2 in HCFs Fig. 4F).

### Effect of signalling pathway inhibitors on cAMP and cGMP signalling activated by ML290 in human primary vascular cells

**Figure 5:**
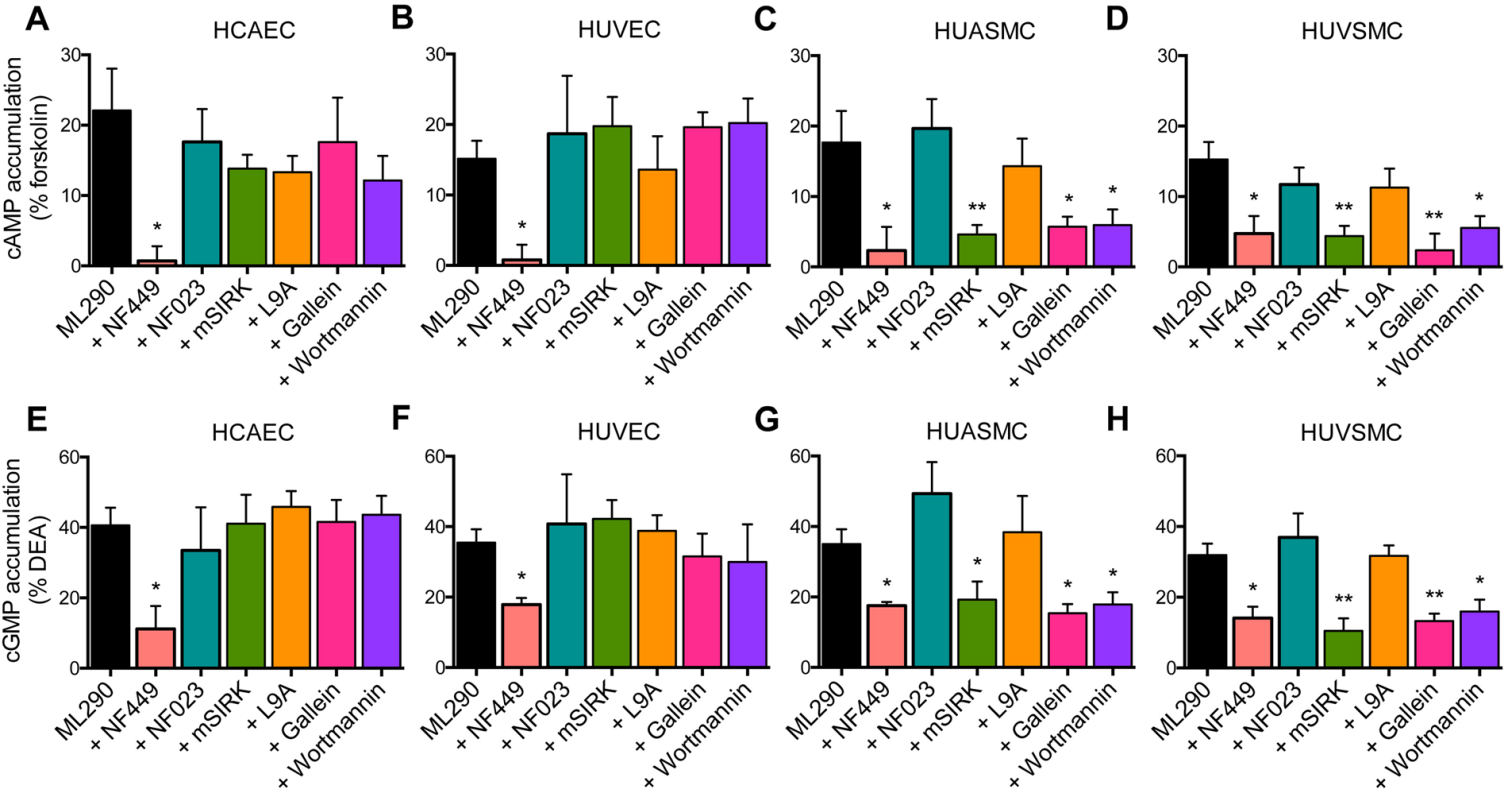
The role of G proteins, βγ subunits and PI3-kinase in ML290-mediated cAMP and cGMP accumulation in human primary vascular cells. ML290 (10 μM, 30 min) increased cAMP accumulation in HCAECs ((**A**) n = 5), HUVECs ((**B**) n = 8), HUASMCs ((**C**) n = 8) and HUVSMCs ((**D**) n = 4). Treatment with the Gαs inhibitor NF449 (10 μM, 30 min) of HCAECs ((**A**) n = 3) and HUVECs ((**B**) n = 4) abolished ML290-mediated cAMP accumulation (30 min) whereas in HUASMCs ((**C**) n = 4) and HUVSMCs ((**D**) n = 4) it reduced the maximum cAMP response. The Gαi/Gα_OB_ inhibitor NF023 (10 μM, 30 min) in HCAECs ((**A**) n = 4) and HUVECs ((**B**) n = 4), HUASMCs ((**C**) n = 4) and HUVSMCs ((**D**) n = 4) had no effect on ML290-mediated cAMP accumulation. Pre-treatment of HCAECs ((**A**) n = 3) and HUVECs ((**B**) n = 3) with the Gβγ inhibitors mSIRK (5 μM, 30 min) and gallein (50 μM, 45 min) and the PI3-kinase inhibitor Wortmannin (100 nM, 30 min) had no effect on ML290-mediated cAMP accumulation whereas pre-treatment of HUASMCs ((**C**) n = 3) or HUVSMCs ((**D**) n = 3) with both Gβγ inhibitors and the PI3-kinase inhibitor reduced the maximum cAMP response to ML290. ML290 (10 μM, 30 min) also increased cGMP accumulation in HCAECs ((**E**) n = 3), HUVECs ((**F**) n = 4), HUASMCs ((**G**) n = 3) and HUVSMCs ((**H**) n = 3). NF449 (10 μM, 30 min) pre-treatment of HCAECs ((**E**) n = 4); HUVECs ((**F**) n = 3), HUASMCs ((**G**) n = 6) and HUVSMCs ((**H**) n = 4) reduced the maximum ML290-mediated cGMP response. Pretreatment with NF023 (10 μM, 30 min) of HCAECs ((**E**) n = 3), HUVECs ((**F**) n = 3), HUASMCs ((**G**) n = 4) and HUVSMCs ((**H**) n = 4) had no effect on ML290-mediated cGMP accumulation. Pretreatment with the Gβγ inhibitors mSIRK (5 μM, 30 min) and gallein (50 μM, 45 min) and the PI3-kinase inhibitor Wortmannin (100 nM, 30 min) had no effect on cGMP accumulation in HCAECs ((**E**) n = 3) and HUVECs ((**F**) n = 3) whereas in HUASMCs ((**G**) n = 5) and HUVSMCs ((**H**) n = 4), it reduced the maximum response. Pre-treatment of HUASMCs ((**C**,**G**) n = 4–11) and HUVSMCs ((**D**,**H**) n = 4–5) with mSIRK control peptide L9A (5 μM, 30 min) had no significant effect on ML290-mediated cAMP or cGMP accumulation. Statistical significance was assessed using a one-way ANOVA with a Dunnet’s post-hoc test compared to ML290 alone: **p < 0.01 and *p < 0.05. Data shown are mean ± SEM of ‘n’ independent experiments.

We next examined the effects of specific inhibitors of G proteins and signalling pathways activated by ML290 associated with cAMP and cGMP accumulation in human vascular cells. We utilized the Gα_s_ inhibitor (NF449), the Gα_i/o_ inhibitor (NF023), the βγ inhibitors (mSIRK and gallein) and the PI3K inhibitor (Wortmannin). In endothelial cells, NF449 (10 μM, 30 min) reduced ML290-mediated cAMP accumulation (% response to 50 μM forskolin) in HCAECs (Figs 5A and S3A) from 22.5 ± 5.5% to 0.8 ± 3.9% (p < 0.05) and in HUVECs (Figs 5B and S3B) from 16.4 ± 2.5% to 0.8 ± 5.7% (p < 0.05). In these endothelial cells, ML290-mediated cAMP accumulation was unaffected by NF023 (10 μM, 30 min), mSIRK (5 μM, 30 min), gallein (50 μM, Fig. 5A,B) or Wortmannin (100 nM, 30 min, Fig. 5A,B), suggesting that ML290-mediated cAMP signalling in these cells was predominantly regulated by Gα_s_.

In contrast, in smooth muscle cells, NF449 (10 μM, 30 min) only partially but significantly inhibited ML290-mediated cAMP accumulation in HUASMCs (Figs 5C and S3C) from 18.8 ± 4.3% to 6.0 ± 7.3% (p < 0.05) and in HUVSMCs (Figs 5D and S3D) from 18.8 ± 8.0% to 4.7 ± 2.5% (p < 0.05). Unlike endothelial cells, in smooth muscle cells, mSIRK (5 μM, 30 min, Figs 5C,D and S3C,D) reduced ML290-mediated cAMP accumulation in HUASMCs from 18.8 ± 4.3% to 9.1 ± 3.3% (p < 0.05) and HUVSMCs from 18.8 ± 8.0% to 3.9 ± 2.5% (p < 0.05). Another βγ inhibitor, gallein (50 μM, Fig. 5C,D), produced similar results to mSIRK reducing ML290-mediated cAMP accumulation in HUASMCs from 16.7 ± 3.4% to 5.2 ± 1.4% (p < 0.05) and in HUVSMCs from 12.6 ± 2.4% to 3.2 ± 3.4% (p < 0.05). Since PI3K is downstream of βγ subunits^15^, we used Wortmannin (100 nM, 30 min, Fig. 5C,D) to determine its role in ML290 signalling and showed inhibition of ML290-mediated cAMP accumulation in HUASMCs from 16.7 ± 3.4% to 7.2 ± 2.2% (p < 0.05) and HUVSMCs from 12.6 ± 2.4% to 6.4 ± 2.2% (p < 0.05). In these cells, like endothelial cells, pre-treatment with the Gi/o inhibitor NF023 (10 μM, 30 min, Figs 5C,D and S3C,D) had no effect on ML290-mediated cAMP accumulation in HUASMC and HUVSMC.

H2 relaxin-mediated cGMP accumulation in HUVECs, HUVSMCs and HUASMCs is known to be mediated by Gα_s_, Gα_i/o_ and PI3K^6^. Using pathway-specific inhibitors in endothelial cells, we found that NF449 significantly reduced the maximum response to ML290 in HCAECs (Figs 5E and S3E) from 41.0 ± 4.5% to 11.9 ± 5.3% and HUVECs (Figs 5F and S3F) from 39.5 ± 4.8% to 22.3 ± 3.0% of the response to DEA. However, ML290-mediated cGMP accumulation was not significantly affected by NF023 (10 μM, 30 min; Figs 5E,F and S3E,F), mSIRK (5 μM, 30 min; Figs 5E,F and S3E,F), gallein (50 μM, Fig. 5E,F) or Wortmannin (100 nM, 30 min, Fig. 5E,F) suggesting that ML290-mediated cGMP signalling, like cAMP signalling, in human primary endothelial cells was predominantly regulated by Gα_s_.

In smooth muscle cells, NF449 (10 μM, 30 min) also reduced ML290-mediated cGMP accumulation in HUASMCs (Figs 5G and S3G) from 41.9 ± 4.8% to 24.3 ± 5.7% (p < 0.05) and in HUVSMCs (Figs 5H and S3H) from 31.4 ± 4.8% to 18.8 ± 3.7% (p < 0.05). In contrast to cGMP responses in endothelial cells, mSIRK (5 μM, 30 min, Figs 5G,H and S3G,H) reduced ML290-mediated cGMP accumulation in HUASMCs from 41.9 ± 4.8% to 20.1 ± 3.9% (p < 0.05) and in HUVSMCs from 31.4 ± 4.8% to 13.4 ± 3.5% (p < 0.01). Gallein (50 μM, Fig. 5G,H) had similar effects to mSIRK and inhibited ML290-mediated cGMP accumulation in HUASMCs from 32.1 ± 4.2% to 15.6 ± 2.6% (p < 0.05) and HUVSMCs from 30.4 ± 3.2% to 14.3 ± 3.8% (p < 0.05). Wortmannin (100 nM, 30 min, Fig. 5G,H) also inhibited ML290-mediated cGMP accumulation in HUASMCs from 32.1 ± 4.2% to 21.8 ± 3.4% (p < 0.05) and HUVSMCs from 30.4 ± 3.2% to 14.3 ± 3.3% (p < 0.05). However, NF023 (10 μM, 30 min, Figs 5C,D and S3C,D) pre-treatment had no effect on ML290-mediated cGMP accumulation in HUASMC and HUVSMC suggesting that as for cAMP accumulation Gα_i/o_ was not involved.

### Chronic ML290 promotes MMP-2 expression and inhibits TGF-β1-induced Smad2 and Smad3 phosphorylation in HCFs

To evaluate potential anti-fibrotic effects of ML290 and compare it to the known effects of H2 relaxin we examined the effects of both mediators on MMP-2 expression and Smad2 and Smad3 phosphorylation (targets of the anti-fibrotic actions of H2 relaxin; refs 20 and 21) in fibrosis-producing HCFs.

**Figure 6:**
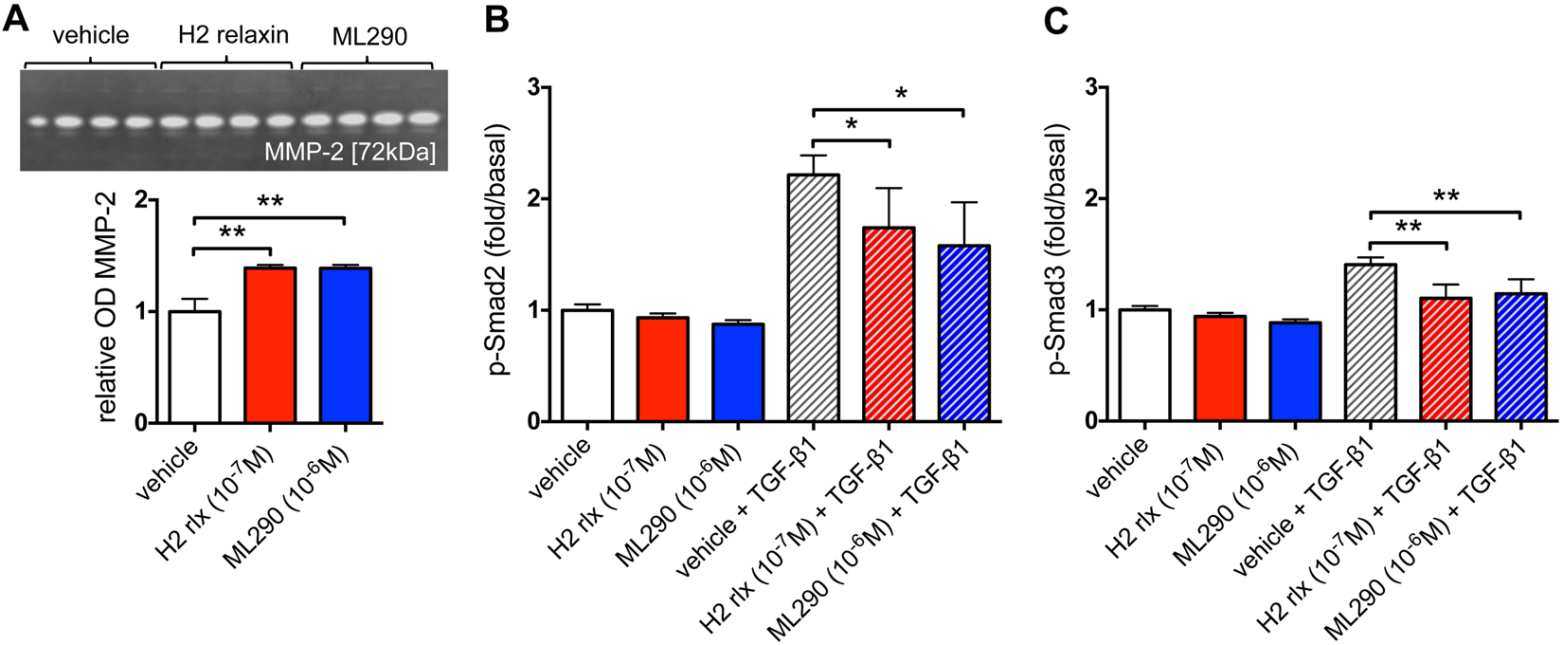
ML290 effects on MMP-2 expression and TGF-β1-induced Smad2 and Smad3 phosphorylation in HCFs. ML290 (1 μM) promoted MMP-2 activity to an equivalent extent to H2 relaxin (0.1 μM) over 72 hours. In (**A** upper) a representative cropped zymograph (see Figure S4) of duplicate samples from two separate experiments; in (**A** lower) mean ± SE OD MMP-2, expressed as the ratio of that of in the untreated control group. **p < 0.01 versus untreated control group. In HCFs treated for 72 hours, ML290 (1 μM) inhibited TGF-β1-induced increases in p-Smad2 (**B**, n = 4) and increases in p-Smad3 (**C**, n = 4) to a level similar to that with H2 relaxin (0.1 μM). TGF-β1 (2 ng/ml) increased p-Smad2 (**B**) and p-Smad3 (**C**) compared to vehicle treated samples. Neither H2 relaxin (0.1 μM) nor ML290 (1 μM) had a significant effect on p-Smad2 or p-Smad3 in cells not treated with TGF-β1 (**B**,**C**). Data are mean ± SEM of 4 independent experiments performed in duplicate, **p < 0.01 and *p < 0.05.

H2 relaxin positively regulates one the main collagen-degrading MMPs, MMP-2, in HCFs^6^. Here, we have investigated the effects of ML290 on MMP-2 in the same cells under the same experimental conditions (Fig. 6A). ML290 (1 μM) increased MMP-2 expression in HCFs by ·40%, as did recombinant H2 relaxin (0.1 μM) when given over 72 hours in culture (both p < 0.01 versus untreated controls).

H2 relaxin, acting on RXFP1, inhibits aberrant myofibroblast differentiation and collagen deposition by disrupting the TGF-β1/Smad2^21^ and TGF-β1/Smad3^20^ axis. In TGF-β1-stimulated HCFs, both H2 relaxin (0.1 μM) and ML290 (1 μM) significantly decreased p-Smad2 and p-Smad3 levels (Fig. 6B,C). Cells treated with TGF-β1 (2 ng/ml) showed a 2.21 ± 0.17-fold increase in p-Smad2 and 1.41 ± 0.07-fold increase in p-Smad3 compared to vehicle-treated samples. Both H2 relaxin (p-Smad2: 1.74 ± 0.13-fold/basal, p < 0.05; p-Smad3: 1.11 ± 0.04-fold/basal, p < 0.01) and ML290 (p-Smad2: 1.58 ± 0.34-fold/basal, p < 0.05; p-Smad3: 1.15 ± 0.05-fold/basal, p < 0.01) significantly inhibited the TGF-β1-induced increase in p-Smad2 and p-Smad3 in HCFs. In contrast, in HCFs not treated with TGF-β1, neither H2 relaxin (0.1 μM) nor ML290 (1 μM) significantly affected p-Smad2 or p-Smad3 levels (Fig. 6B,C).

## Discussion

**Figure 7:**
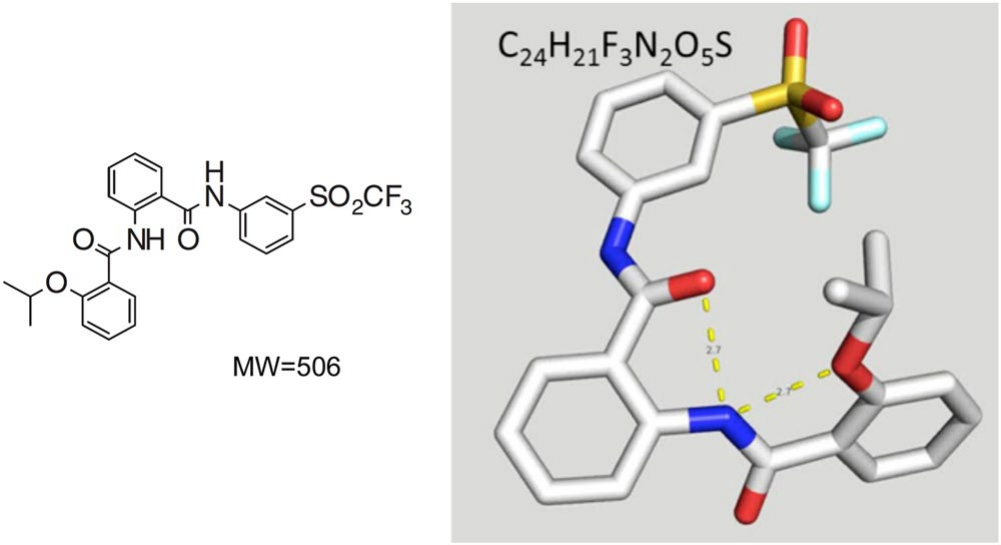
Chemical structure of ML290^13^. 2-Isopropoxy-N-(2-(3-(tri fluoromethyl sulfonyl) phenyl carbamoyl) phenyl) benzamide.

ML290 was discovered by high throughput screening of >350,000 small molecules followed by lead optimisation (Fig. 7)^13^. ML290 was reported to have selectivity for RXFP1, high nanomolar potency in cAMP assays and did not compete with H2 relaxin binding. Interaction with an allosteric site was considered likely because of the complex mode of interaction of H2 relaxin with the orthosteric binding sites on RXFP1. This involves the binding of H2 relaxin with high affinity to the leucine rich repeat (LRR)^22, 23^ and to the LRR-LDLa linker region^24^ in the ectodomain of RXFP1 such that binding to the latter region stabilizes and extends a helical conformation within the linker that acts as the critical switch for LDLa-mediated receptor activation^24^. Thus for signalling involving the H2 relaxin peptides, the LDLa module is essential and behaves as a tethered ligand to activate RXFP1 via interactions at an allosteric activation site on the TM domain^24, 25^. Receptors that lack the LDLa module or contain mutations that disrupt its structure^26, 27^ or the predicted functional amino acids that drive activation^27, 28^ bind H2 relaxin with high affinity but do not signal. However, and in contrast to H2 relaxin, ML290 still activates cAMP production with LDLa mutant receptors^13, 24^ or with an LDLa-less receptor (data not shown) suggesting a different mode of action.

The agonist actions of ML290 are also species-specific being confined to the human, monkey and pig RXFP1 with antagonist actions at the mouse^11, 14^. These actions are interesting as it suggests that ML290 interacts with a binding site that overlaps with that thought to be activated by the LDLa module. Mutation and modelling studies demonstrate that the site is located within a binding pocket formed by the TM domains with ML290 mainly interacting with a hydrophobic patch W664/I667/F668 located at the extracellular end of TM7 and with G659/T660 in ECL3^11, 13^. The interaction with ECL3 is particularly important for the specificity of action of ML290 at RXFP1. Differences in G659/T660 in RXFP2 or across species RXFP1 determine whether ML290 produces the conformational changes required for an agonist action^11, 13^. These properties together with the initial evidence that ML290 does not directly compete for ^125^I-H2 relaxin binding at the orthosteric binding sites on human RXFP1 suggests an allosteric mode of action. The current approach provided additional supporting evidence for this view from detailed binding studies. We found that ML290 does not alter the affinity of H2 for RXFP1 in competition studies (Fig. 1B), producing instead a concentration-dependent small potentiation in binding of ^125^I-H2 relaxin toward RXFP1 (Fig. 1A) possibly mediated by conformational changes induced by binding to the allosteric site. Examination of the kinetics of binding showed that ML290 did not affect either the rate of association or dissociation of ^125^I-H2 relaxin at RXFP1 thus presenting ML290 as an agonist acting at a topographically distinct allosteric activation site that has minimal effects on the binding of H2 relaxin. The data is consistent with the LDLa module being a tethered ligand that interacts with the TM domain to initiate activation. It is possible that the binding site of ML290 is in close apposition to the LDLa interaction site.

Previous studies have used PTX-resistant variants of G proteins to establish that RXFP1 couples to Gα_s_ to increase cAMP, an effect that is negatively modulated by coupling to Gα_OB_^15^. There is also coupling to Gα_i3_^15, 29, 30^ that results in activation of PI-3-kinase and translocation of PKCζ to produce delayed stimulation of adenylyl cyclase V to cause a late surge of cAMP. In the present studies we have examined coupling to G proteins using BRET to directly compare the patterns produced by H2 relaxin and ML290. Both H2 relaxin and ML290 produced changes in the BRET ratio between RXFP1 and Gα_s_ or Gα_i3_ that were quantitatively and qualitatively similar (Fig. 2A,C). However the pattern of BRET ratios between RXFP1 and Gα_OB_ produced by H2 relaxin and ML290 were clearly different (Fig. 2B) suggesting that these ligands cause different conformational changes in the receptor.

The pattern of G protein coupling was also examined in human primary vascular cells using the specific G-protein inhibitors, NF449 (Gα_s_)^31^, NF023 (Gα_i/o_)^32^ and mSIRK (Gβγ)^33^ for cAMP and cGMP signalling. The results were in broad agreement with those using BRET in HEK cells and confirmed that Gα_s_ was the major G-protein involved although again there were cell type differences. Thus, in endothelial cells, cAMP and cGMP responses were blocked by NF449 but not affected by NF023 or mSIRK whereas in smooth muscle cells both NF449 and mSIRK reduced responses. Another Gβγ inhibitor gallein^34^ produced the same effects as mSIRK and the control peptide L9A had no effect. The lack of effect of NF023 on cAMP or cGMP accumulation may be related to blockade of both inhibitory Gα_OB_ and stimulatory Gα_i3_ that would tend to cancel out any change. It is likely that the effects downstream of Gβγ involve PI-3-kinase since the PI-3-kinase inhibitor Wortmannin produced a similar pattern to gallein.

**Figure 8:**
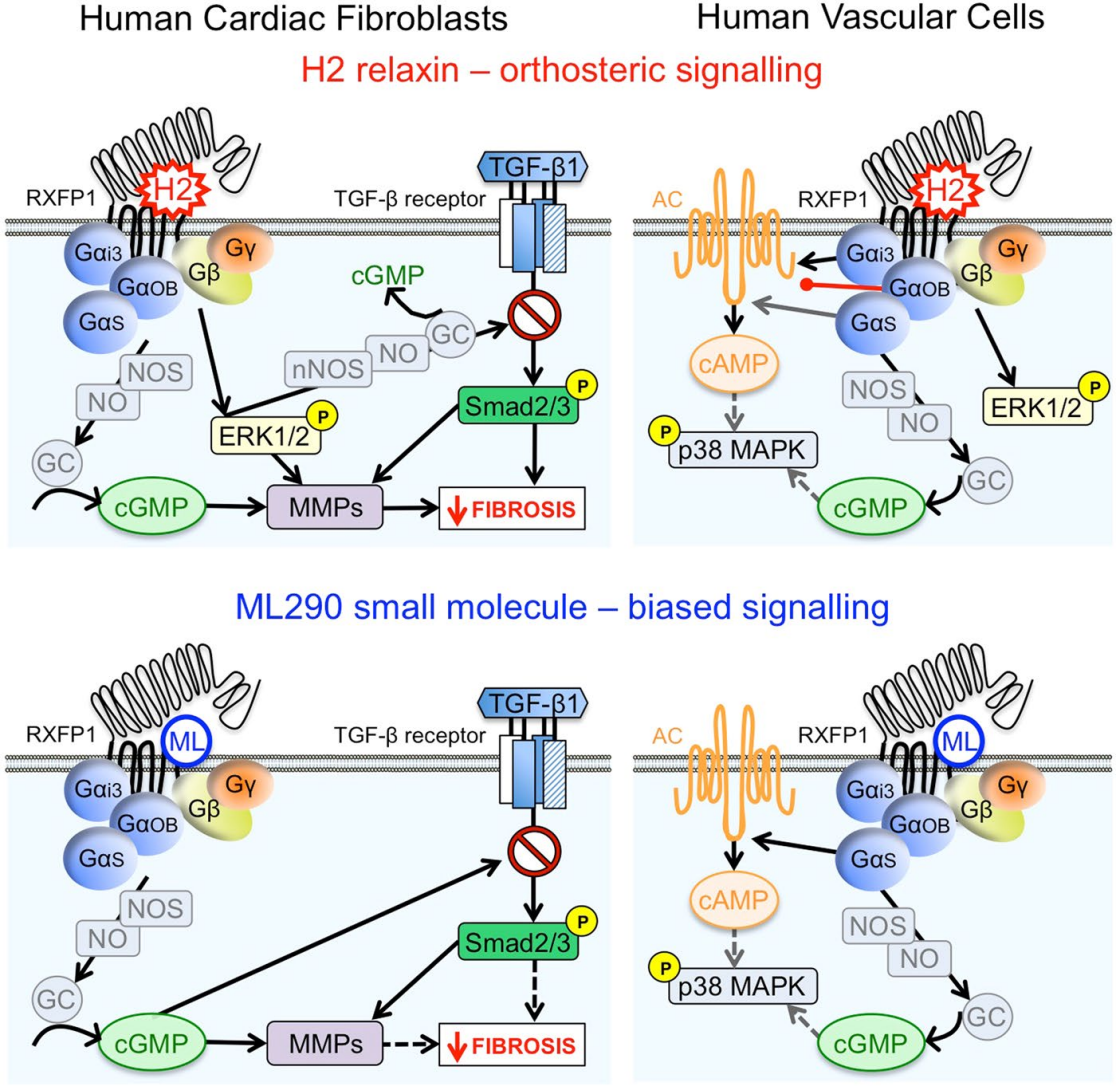
Comparison of signalling pathways activated by ML290 and H2 relaxin through RXFP1 expressed in human primary vascular cells and myofibroblasts. In human primary myofibroblasts both H2 relaxin and ML290 have a profile corresponding to anti-fibrotic activity yet ML290 achieves this without activating ERK. In human primary vascular cells H2 relaxin activates cAMP, cGMP and pERK with similar potency whereas ML290 is biased towards cGMP signalling compared with cAMP and does not activate pERK.

Comparison of the signalling pathways that were activated by H2 relaxin and ML290 in HEK-RXFP1 cells showed that while both H2 relaxin and ML290 caused increases in cAMP and p-p38MAPK only H2 relaxin activated p-ERK1/2 (Fig. 8), and neither ligand activated p-JNK1/2/3. Previous studies have shown that the PKA inhibitor H89 inhibited p38MAPK phosphorylation in response to relaxin in HEK-RXFP1 cells^35^. Since H2 relaxin does not promote internalisation, it is unlikely that the lack of a pERK1/2 response to direct application of ML290 results from a lack of β-arrestin recruitment and internalisation. Our findings show that in addition to acting allosterically, ML290 displayed signalling bias showing similar potency to H2 relaxin for activation of p-p38MAPK, lower potency than H2 relaxin for cAMP generation and unlike H2 relaxin being ineffective in activating pERK1/2. The enhancement of the pERK response to relaxin by ML290 was interesting and indicated that ML290 does not directly occupy the activation site utilised by the LDLa module at the human RXFP1. Rather it resembles the small enhancement of ^125^I H2 relaxin binding seen with ML290 suggesting that this could be responsible and both effects could result from a change in the conformation that RXFP1 can adopt when the allosteric site is occupied. Further studies will be required to clarify this point.

Since there is particular interest in the cardiovascular effects of H2 relaxin in humans, we examined signalling pathways in human primary vascular cells. Previous studies show that H2 relaxin activates cAMP, cGMP and ERK1/2 signalling (Fig. 8) in human endothelial and smooth muscle cells^6^ and that the cGMP response was greatly enhanced in co-cultures of endothelial and smooth muscle cells^17^. Like H2 relaxin, ML290 produced cAMP and cGMP responses in HCAEC, HUVEC, HUASMC, HUVSMC (Fig. 8) but not in HUAEC that have been shown to lack cell surface expression of RXFP1^6^. A particularly interesting observation was that the concentration-response relationship for cGMP responses produced by ML290 was shifted to the left by more than an order of magnitude compared to that for cAMP (Fig. 4). In addition, ML290 produced p-p38MAPK responses only in smooth muscle and not in endothelial cells and in none of the cell types was there a pERK1/2 response recorded to direct addition of ML290.

Comparison of the patterns of signalling to ML290 in recombinant and human primary cell systems showed that cAMP signalling was similar as was the lack of a p-ERK1/2 response (Fig. 8). However, in HEK-RXFP1 cells, both ML290 and H2 relaxin failed to cause cGMP accumulation (data not shown) whereas it was a dominant response in human primary vascular cells, suggesting that the cellular background has a significant role to play in determining the signal transduction mechanisms observed with both H2 relaxin and ML290. p-p38MAPK responses were only observed in HEK cells and human smooth muscle cells. Thus, not only is there signalling bias displayed by ML290 there is also tissue bias that influences the signalling pathways observed.

The effect of ML290 on cAMP and cGMP may be clinically relevant since ML290 shows bias towards cGMP signalling in human primary vascular cells that suggests that ML290 may be a vasodilator. cGMP regulates vascular tone by regulating cytosolic Ca^2+^ levels in smooth muscle cells, Ca^2+^ sensitivity of myofilaments and smooth muscle cell proliferation and differentiation^36–38^. ML290 also stimulated cGMP signalling in endothelial cells (Fig. 4B) that contribute to angiogenesis^38^ a role supported by evidence that ML290 increases VEGF expression^13^. ML290 also activated cAMP signalling in human primary vascular cells, albeit with a lower potency than cGMP. cAMP has conflicting roles, being pro-arrhythmic and producing increases in heart rate and intracellular Ca^2+^ levels, factors that may be detrimental to myocardial infarction and heart failur^39, 40^. However there may also be beneficial effects since transgenic mice with increased cardiac AC_VI_ expression that underwent acute myocardial infarction showed an increase in cAMP levels but reduced left ventricular remodelling and mortality^41^. Therefore, the role of ML290 in cardiac (patho)physiology will be important to determine.

Another important finding was that in contrast to H2 relaxin^6^, direct addition of ML290 failed to cause ERK1/2 activation (Table 1 and Figs 3, 4 and 8). ERK1/2 signalling plays an important role in cardiac hypertrophy^42^ a compensatory mechanism accompanying heart failure that is beneficial in the early stages but later leads to failure^42^. Since ERK and other MAPKs such as p38MAPK and JNK also play important roles in cardiomyocyte survival^42^ and can promote TGF-β1 signal transduction and cardiac fibrosis^43^, the pathophysiological relevance of the lack of ERK1/2 activation (Figs 3 and 4) and cell-dependent p38MAPK phosphorylation (Figs 3 and 4) by ML290, and the interaction of relaxin and ML290 on pERK1/2 signalling will need further investigation.

Comparison of the bias profile of ML290 with the recently described functionally selective single chain relaxin derivative B7-33^44^ reveals some important differences. Unlike ML290, B7-33 competes with relaxin binding at the orthosteric site and promotes cAMP accumulation following stimulation of rat and mouse RXFP1 as well as human RXFP1 albeit with 3–5 orders of magnitude lower potency than H2 relaxin^44^. Like ML290, B7-33 has little if any activity at RXFP2^44^. In contrast to ML290, that displayed no ERK1/2 activation in any system tested, B7-33 is a strong activator of ERK1/2 in rat renal myofibroblasts with a potency and efficacy comparable with H2 relaxin^44^. B7-33 and H2 relaxin increase MMP2 activity by similar mechanisms^44^ whereas the direct effect of ML290 is not associated with ERK1/2 activation.

The anti-fibrotic actions of H2 relaxin, including its ability to inhibit the TGF-β1/p-Smad2 axis and TGF-β1-induced myofibroblast differentiation and collagen deposition, while promoting collagen-degrading MMPs, have been shown to be mediated through RXFP1-ERK1/2 signalling in HCFs^6^, human dermal fibroblasts^45^ and rat renal myofibroblasts^46–48^. In these studies, it was suggested that H2 relaxin stimulated cGMP accumulation down-stream of its ability to activate p-ERK1/2 through a RXFP1-p-ERK1/2-nNOS-NO-sGC-cGMP-dependent pathway because: (i) H2 relaxin stimulated pERK1/2 and cGMP within minutes of administration to (myo)fibroblast cultures, but it required 48–72 hours to stimulate nNOS expression in HCFs and rat renal myofibroblasts, and these effects were abrogated by the MEK inhibitor, PD98059^46^; (ii) the inhibition of the TGF-β1/p-Smad2 axis and α-SMA expression (used as a marker of myofibroblast differentiation) by H2 relaxin over 72 hours was blocked by the nNOS inhibitor, N-propyl-L-arginine, or the sGC inhibitor ODQ; and (iii) sGC is the only known receptor for NO that stimulates cGMP accumulation, leading to the proposal that cGMP is acting downstream of NO-sGC. The finding that ML290 rapidly stimulated cGMP accumulation without ERK1/2 activation, and independently that H2 relaxin-induced stimulation of cGMP was significantly inhibited by co-administration of ODQ^48^ may indicate that cGMP may be involved with both ligands but by different mechanisms. Both ligands appear capable of directly causing activation of sGC and cGMP accumulation but only H2 relaxin can activate the RXFP1-p-ERK1/2-nNOS-NO-sGC-dependent pathway. H2 relaxin, B7-33 and ML290 have distinctly different signalling profiles and will be useful tools to determine the relative importance of these profiles in the treatment of cardiac failure and the anti-fibrotic effect. Further studies will be required to verify the contributions of the mechanisms involved.

H2 relaxin has recently completed an extended phase III clinical trial for the treatment of AHF having previously been shown to improve dyspnoea and 180-day survival^2^. Although it has consistently been shown to be safe, H2 relaxin has a number of disadvantages. The peptide displays cross-reactivity with other relaxin family peptide receptors^9^, is not orally bioavailable and has a short half-life (<10 min)^10^. Thus, potent, selective small molecule agonists of RXFP1 may have additional benefits, including oral bioavailability, a longer duration of action and a more flexible treatment protocol. The biased signalling profile of ML290 with strong activation of cGMP signalling in vascular cells suggests that ML290 will display vasodilator and organ protective properties whereas the lower potency for cAMP generation will minimise effects on VEGF expression and consequent angiogenesis. The discovery of an allosteric biased small molecule agonist for RXFP1, with a half-life of several hours^11, 13^, could have important implications for the treatment of AHF.

Relatively little has been published on other properties of ML290. However, it is known to have little or no action at RXFP2, RXFP3 and AVPR1B, little cytotoxicity and to have high microsomal stability. The half-life in plasma and heart is long (T_1/2_ 8.56 and 7.48 h) and ML290 is concentrated in the heart^13^. While ML290 increased VEGF expression in THP-1 cells it would be of interest to examine the longer-term effects in an *in vivo* model on MMPs, tissue inhibitors of MMPs (TIMPs) and NOS to confirm potential anti-fibrotic properties. However, given the species selectivity of ML290 development of an *in vivo* model will be challenging^14^. A better understanding of the effects of ML290 on NOS and ET_B_ expression would also further enhance our knowledge of the mechanisms underlying potential vasodilatory effects of ML290. Production and testing of mice with humanized RXFP1 might resolve these questions. It is of paramount importance to determine if the findings seen in our study can be translated to *in vivo* humanized mouse models .

We have provided direct evidence that the first small molecule agonist at RXFP1, ML290, is an allosteric biased agonist at RXFP1 that preferentially increases cGMP accumulation relative to cAMP in human vascular cells and does not directly activate p-ERK1/2 in any of the systems studied. ML290 increased p-p38MAPK but only in human primary smooth muscle cells and not in endothelial cells. Examination of the pattern of G protein coupling in both recombinant and primary cell systems indicated that ML290 resembles relaxin in terms of the conformational changes associated with Gα_s_ coupling but is quite different with regard to Gα_OB_ coupling. The signalling bias observed with ML290 towards cGMP and away from cAMP and particularly pERK1/2 suggests vasodilator and antifibrotic actions while minimising effects on angiogenesis and hypertrophy.

## Materials and Methods

### Materials

Human gene 2 relaxin (H2 relaxin) was supplied by Corthera Inc. (San Mateo, CA, USA; a subsidiary of Novartis AG, Basel, Switzerland) and ML290 by National Center for Advancing Translational Sciences, National Institutes of Health, Maryland, USA. NF023, NF449, mSIRK and L9A were purchased from Calbiochem (Australia); gallein, pertussis toxin and forskolin from Sigma (Australia); foetal bovine serum (FBS) from JRH Biosciences (KS, USA); coelenterazine *h* from Promega (WI, USA); geneJuice from Merck (Victoria, Australia); and DEA NONOate from Cayman Chemicals (NSW, Australia).

### Constructs

G protein cDNA clones Gβ1 and Gα subunits Gα_s_, Gα_oB_, Gα_i3_ were from Missouri S&T cDNA Resource Cente (MO, USA) and Gγ2-Venus was kindly supplied by Prof Michel Bouvier (Université de Montréal, Montréal, Quebec, Canada).

### Cloning of RXFP1-Rluc8

A mammalian expression plasmid containing RXFP1-Rluc8 was produced by modifying a pcDNA3.1 RXFP1-GFP2 plasmid (Svendsen *et al*., 2008). Briefly the GFP2 sequence was removed from the plasmid utilizing engineered NotI and XhoI cut sites. The Rluc8 sequence was then amplified from pcDNA V2R Rluc8 by RT-PCR using specific primers flanked by NotI and XhoI sites (Supplementary Table S1). This PCR product was then cut with NotI and XhoI followed by ligation into the pcDNA3.1 RXFP1-GFP2 plasmid to create pcDNA3.1 RXFP1-Rluc8. The final plasmid insert was sequenced on both strands to ensure there were no errors in the PCR amplification or cloning process.

### Generation of cell lines stably expressing RXFP1

A lentiviral construct containing RXFP1-Rluc8 under the control of the Ef1α promoter (pLenti6 Ef1α RXFP1-Rluc8) was generated using the multisite Gateway cloning system (Invitrogen)^49^. Briefly, RXFP1-Rluc8 was generated using RT-PCR with primers containing the required sequences at recombination sites in addition to 18–25 base pairs of template-specific sequence from pcDNA3.1 RXFP1-rluc8 (Supplementary Table S1). The RXFP1-rluc8 PCR product was inserted into the pDONR 221 P5-P2 backbone vector utilizing the BP ClonaseII enzyme (Invitrogen) to create the pENTR L5-L2 RXFP1-Rluc8 entry vector. To generate the final pLenti6 Ef1α RXFP1-RlUC8, plasmid pENTR L5-L2 RXFP1-Rluc8 and pENTR L1-R5 Ef1α (kind gift of Dr Melanie White, Monash University) were added to the destination vector pLenti6-Blockit-DEST and Clonase II Plus enzyme mix (Invitrogen). The final insert was sequenced on both strands to ensure there were no errors in the PCR amplification or cloning process.

Lentiviruses were generated by co-transfection of 80% confluent HEK293T cells using Lipofectamine 2000 transfection reagent (Invitrogen, Life Technologies) with pLenti6 Ef1α RXFP1-Rluc8 and the packaging plasmids (pMDL, pRev and pVSVG; Kind gifts of Prof Alon Chen, Weizmann Institute of Science, Israel) which provide the required trans-acting factors, namely Gag-Pol, Rev and the envelope protein VSVG, respectively^50^. Lentivirus secreted into the media was collected and passed through a 30 mm diameter 0.45 μm Durapore PVDF syringe filter (Millipore) to remove any cell particulates prior to storage at −80 °C. For the production of stable cells expressing RXFP1-Rluc8, HEK293T cells seeded on a 10 cm cell culture dish were transduced with the harvested pLenti6 Ef1α RXFP1-Rluc8 recombinant lentivirus by replacing the cell culture media with 10 mL of the virus-containing media, mixed with polybrene (Millipore) to increase the transduction efficiency. Two rounds of 24-hour transduction were performed before the cells were allowed to recover for 48 hours in complete DMEM. The cells were then transferred to 175 cm^2^ flasks and grown to confluency. Transduced cells were FACS sorted (Becton Dickinson FACS AriaIII) utilizing an anti-FLAG antibody to detect the N-terminal FLAG tag on the receptor as previously described and the highest cell surface receptor expressing cells collected^51^. Only the top 10% of cells with fluorescence levels significantly higher than the background fluorescence in non-transfected HEK293T control cells were collected. Sorted cells were propagated in complete DMEM for use in assays.

### Cell Culture

HEK293 parental cells and cells stably expressing RXFP1 (HEK-RXFP1) were cultured in DMEM containing 5% (v/v) FBS, 100 units/ml penicillin, 100 µg/ml streptomycin and maintained at 37 °C under 5% CO_2_. Transient transfections of HEK-RXFP1 cells were carried out 24 h after seeding using GeneJuice (Merck, Kilsyth, Australia) according to the manufacturer’s instructions. Primary cultures of human umbilical artery endothelial cells (HUAEC), human umbilical vein endothelial cells (HUVEC), human coronary artery endothelial cells (HCAEC), human umbilical artery smooth muscle cells (HUASMC) and human umbilical vein smooth muscle cells (HUVSMC) were obtained from ScienCell Research Laboratories (San Diego, U.S.A) and cultured as detailed previously^6^. Human foetal cardiac fibroblasts (HCF) were characterized by the presence of fibronectin (ScienCell). Cells were maintained in Medium 199 containing 5% FBS, 100 units/ml penicillin, 100 µg/ml streptomycin and the relevant growth supplements for optimal growth of each cell type (ScienCell) and maintained at 37 °C under 5% CO_2_. Fibroblasts were grown in fibroblast cell growth supplement-2 (BSA 10 μg/ml, apo-transferrin 10 μg/ml, insulin 7.5 μg/ml, EGF 2 ng/ml, FGF-2 2 ng/ml, hydrocortisone 1 μg/ml) (information provided by ScienCell). Early passage primary cell cultures (2–5) were used for all experiments.

### Radioligand binding

Competition radioligand binding studies were performed in HEK-RXFP1 cells as described previously^23^. Briefly, HEK-RXFP1 cells were incubated with 100pM of ^125^I-H2 relaxin and allowed to compete with increasing concentrations of unlabelled H2 relaxin in the presence of ML290 or vehicle (0.1% DMSO). After 90 min incubation at room temperature, the reaction was terminated with the removal of medium and washing with cold PBS. Cells were digested with 0.1 M NaOH and radioactivity counted on the γ-counter.

### Kinetic studies

Association and dissociation kinetic studies were performed using ^125^I-H2 relaxin. Briefly, HEK-RXFP1 cells were plated in 96-well plates and allowed to adhere overnight. After rinsing with PBS, the cells were incubated at the given time intervals with 100pM of ^125^I-H2 relaxin to measure the association rate. The dissociation rate of ML290 was calculated using ligand competition. Cells were allowed to equilibrate with 100 pM of ^125^I-H2 relaxin for 90 mins prior to the addition of unlabelled 200 nM H2 relaxin for the given time intervals. The reactions were terminated by the removal of medium and washing with ice-cold PBS. Cells were digested using 0.1 M NaOH and radioactivity counted on the γ-counter.

### ERK1/2, p38MAPK or JNK1/2 Phosphorylation Assays

p-ERK1/2, p-p38MAPK and p-JNK1/2 was measured using the Surefire ERK, p38 and JNK kits (TGR BioSciences, Australia) as described previously^6, 16^. Briefly, HEK-RXFP1 cells were plated into 96-well plates (5 × 10^4^ cells/well) and primary cells grown in 24-well plates (1 × 10^5^ cells/well) overnight to achieve a confluent monolayer. Prior to stimulation, HEK293 cells were serum-starved with DMEM containing 0.5% (v/v) FBS and primary cells in M199 medium for 4–6 hours. The effect of ML290 on H2 relaxin-stimulated p-ERK1/2 was examined by addition of ML290 (10^−5^ M or 10^−6^ M) or vehicle (DMSO) to HEK-RXFP1 cells for 10 min at 37 °C followed by H2 relaxin (10^−5^ M–10^−12^ M) and incubation at 37 °C for 4 min (maximal response). Levels of p-ERK1/2, p-p38MAPK and p-JNK1/2/3 were detected as per manufacturer’s instructions (Perkin-Elmer, Australia).

### cAMP and cGMP Accumulation Assays

cAMP and cGMP accumulation was determined as previously described^6, 16^. Briefly, HEK293 cells were plated in 96-well plates (5 × 10^4^ cells/well) and human primary cells were plated into 24-well plates (1 × 10^5^ cells/well) and grown overnight to achieve a confluent monolayer. Prior to stimulation, HEK cells were serum-starved in DMEM containing 0.5% FBS v/v and primary cells were serum-starved in M199 medium for 4–6 hours. Where appropriate, cells were pre-incubated with mSIRK (5 μM, 30 min), mSIRK control peptide L9A (5 μM, 30 min), gallein (100 μM, 30 min), Wortmannin (100 nM, 30 min), NF023 (10 μM, 30 min) and NF449 (10 μM, 30 min). Levels of cAMP and cGMP were detected according to the manufacturer’s instructions (Perkin-Elmer, Australia).

### Gelatin zymography

Changes in matrix metalloproteinases-2 (MMP-2; gelatinase A) activity that were secreted from HCFs into the cell media over a 72 hour experimental period, were assessed by gelatin zymography as described previously^44, 45^. Cells were treated with either ML290 (1 μM), H2 relaxin (0.1 μM; a 10-fold lower concentration based on the cGMP concentration-response curve) or vehicle (0.1% DMSO) for 72 hours and analysed for MMP-2 expression. Cells were used between passages 1–4, while all experiments were performed four separate times in duplicate. The optical density (OD) of MMP-2 was measured using a GS710 Calibrated Imaging Densitomer (Bio-Rad Laboratories, Hercules, CA, USA), and the mean ± SE OD of MMP-2 in each treated group was expressed as the relative ratio of the values in the untreated control group.

### Smad2 and Smad3 Phosphorylation Assays

To evaluate the anti-fibrotic effect of ML290 and compare it to H2 relaxin in HCFs were plated into 24-well plates (0.7 × 10^5^ cells/well) and grown overnight to achieve a confluent monolayer. Cells were treated with either ML290 (1 μM), H2 relaxin (0.1 μM) or vehicle (0.1% DMSO) or in combination with TGF-β1 (2 ng/ml final concentration) for 72 hours. Cells were serum-starved in M199 medium for 24 hours prior sample collection. Cells were used between passages 1–4, while all experiments were performed four separate times in duplicate. Levels of p-Smad2 and p-Smad3 were detected using Sure-fire kits according to the manufacturer’s instructions (Perkin-Elmer, Australia).

### Real-time kinetic BRET assays

HEK293 cell were seeded in 6-well plates at a density of 600,000 cells per well and transfected with constructs encoding the tagged receptors and signalling proteins. 24 h later cells were harvested in HEPES-buffered phenol red-free medium containing 5% FBS and plated out in a white 96-well plate (Nunc) that was incubated at 37 °C under 5% CO_2_. BRET assays were performed 48 h after transfection as described previously^16, 52^. Medium was replaced with phenol-red-free-DMEM supplemented with 5% FBS plus 5 μM coelenterazine *h*. BRET measurements were made at 37 °C using the PHERAstar Omega plate reader with Omega software (BMG LABTECH, Germany). Filtered light emissions were simultaneously measured in each of the “donor wavelength window” (475 ± 15 nm for Rluc8 with coelenterazine *h*) and “acceptor wavelength window” (535 ± 15 nm for Venus). Cells were assayed before and after treatment with ligands or 5% FBS phenol-red-free-DMEM medium containing 0.01% w/v BSA (vehicle). The BRET signal was calculated as previously described^52^.

### Data Analysis

Data was analysed using GraphPad Prism v6.0. All data represents the mean ± S.E.M of ‘*n*’ individual experiments performed in duplicate. Concentration-response curves were fitted either using a sigmoidal or Gaussian model. Kinetic data was plotted using the association and dissociation models to calculate *k*_on_ and *k*_off_. Statistical significance was determined using a Student’s t-test or a one-way ANOVA with a Dunnett’s post-hoc test with significance accepted at p < 0.05.

## Electronic supplementary material

Supplementary File SREP-17-02520
