# Supplementary material for: ML290 is a biased allosteric agonist at the relaxin receptor RXFP1
Source: Sci Rep. 2017 Jun 7;7:2968. doi: 10.1038/s41598-017-02916-5 (PMC5462828; doi:10.1038/s41598-017-02916-5)
Supplement: Supplementary file 1 — Supplementary File SREP-17-02520 [file 41598_2017_2916_MOESM1_ESM.pdf]

ML290 is a biased allosteric agonist at the relaxin receptor RXFP1

Martina Kocan[1#], Mohsin Sarwar[1#], Sheng Y. Ang[1], Jingbo Xiao[2], Juan J. Marugan[2], Mohammed A. Hossain[3], Chao Wang[5], Dana S. Hutchinson[1], Chrishan S. Samuel[5], Alexander I. Agoulnik[6], Ross A.D. Bathgate[3 4] and Roger J. Summers[1]*

[1]Drug Discovery Biology, Monash Institute of Pharmaceutical Sciences, Monash University, Parkville, Australia
[2]Preclinical Innovation, National Center for Advancing Translational Sciences, National Institutes of Health, Maryland, USA
[3]The Florey Institute of Neuroscience and Mental Health, Parkville, Australia
[4]Department of Biochemistry and Molecular Biology, University of Melbourne, Parkville, Australia
[5]Department of Pharmacology, Monash University, Clayton, Australia
[6]Department of Human and Molecular Genetics, Herbert Wertheim College of Medicine, Florida International University, Florida, USA (A.I.A).
#These authors contributed equally to this work.

Supplementary Information

Supplementary Table S1: Primers used for cloning of RXFP1-rLuc8 constructs.

pcDNA3.1 RXFP1-rluc8 Primers
Rluc8 Not I fwd
5' CATCATGCGGCCGCGCTTCCAAGGTGTACGACC 3'
Rluc8 Xho I reverse
5' CATCATCTCGAGTTACTGCTCGTTCTTCAGCAC 3'

pLenti6 Ef1α RXFP1-Rluc8 Primers
Att B5 RXFP1 Fwd: 48mer
5' gggg ACA ACT TTG TAT ACA AAA GTT G ATG GAC AGC AAA GGT TCG TCG C 3'
Att B2 rLuc8 Rev: 52mer
5' gggg AC CAC TTT GTA CAA GAA AGC TGG GTA TTA CTG CTC GTT CTT CAG CAC G 3'

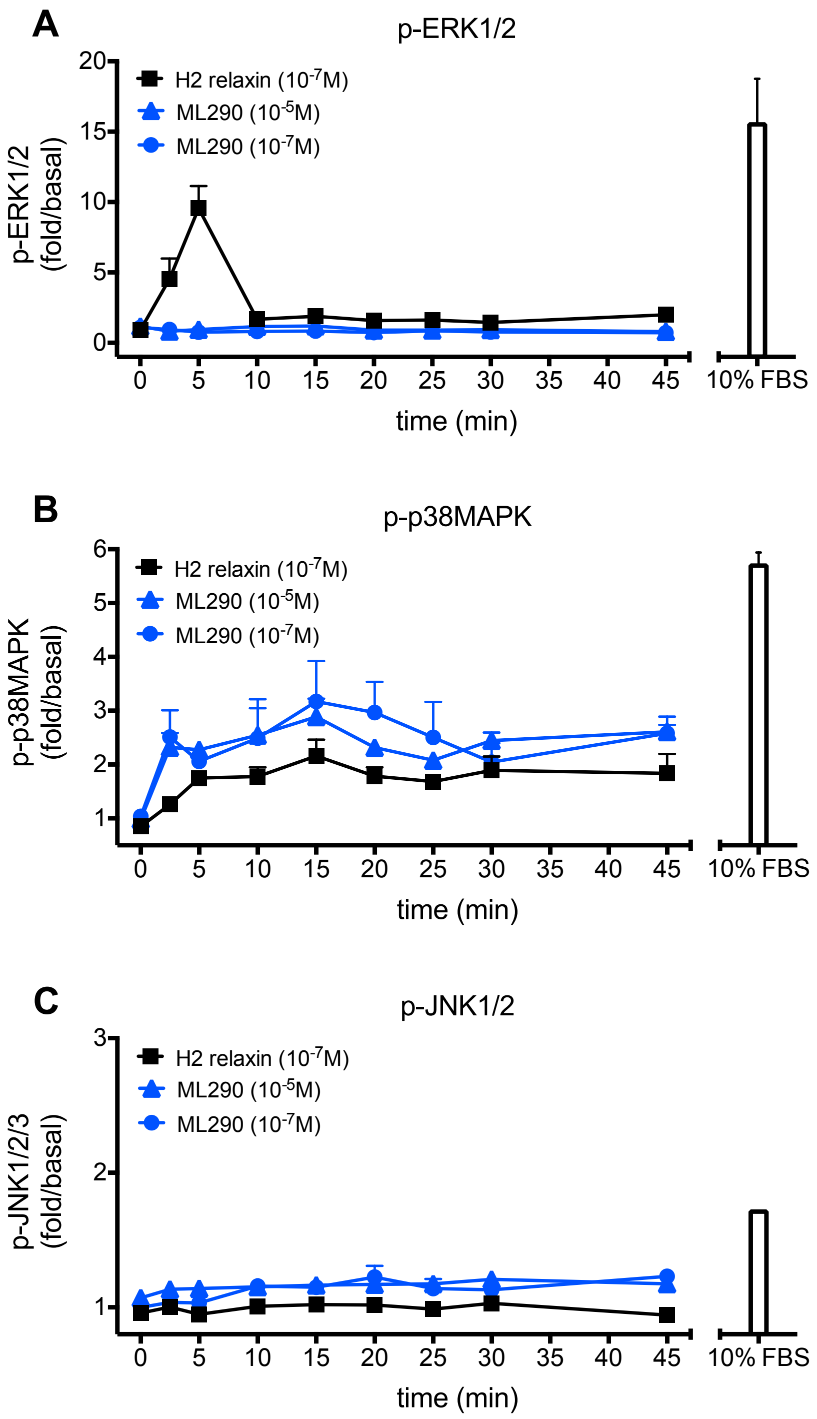

Figure S1 Time course of activation of ERK1/2 (A), p38MAPK (B) and JNK1/2 (C) following addition of H2 relaxin or ML290 in HEK-RXFP1 cells. *Cells were treated for periods of up to 45 min, and p-ERK1/2 (A), p-p38MAPK (B) and pJNK1/2 (C) activation quantified using phospho-"kinase"-specific Surefire AlphaScreen kits. H2 relaxin and ML290 both stimulated p38MAPK but had no effect on JNK1/2/3 phosphorylation. ERK1/2 phosphorylation was activated by H2 relaxin (0.1μM) but not ML290 (0.1μM or 10μM). Data are mean ± SEM for 4 independent experiments.*

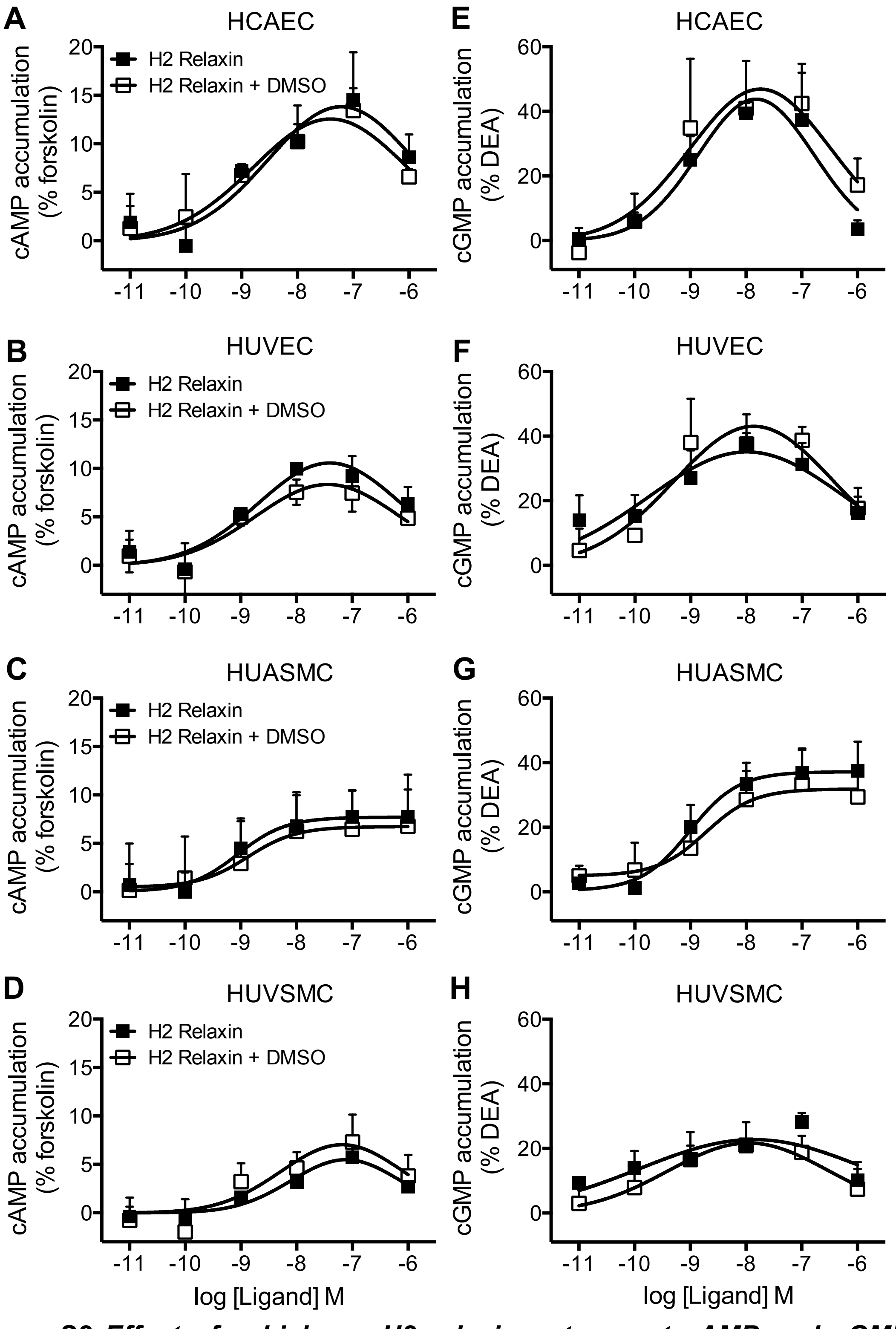

Figure S2 Effect of vehicle on H2 relaxin potency at cAMP and cGMP accumulation in human primary vascular cells. *DMSO (final concentration 1%) had no effect on the potency and efficacy of H2 relaxin (30 min) on cAMP or cGMP accumulation in HCAECs (A,E), HUVECs (B,F), HUASMCs (C,G) and HUVSMCs (D,H). Data shown are mean ± SEM of 3 independent experiments.*

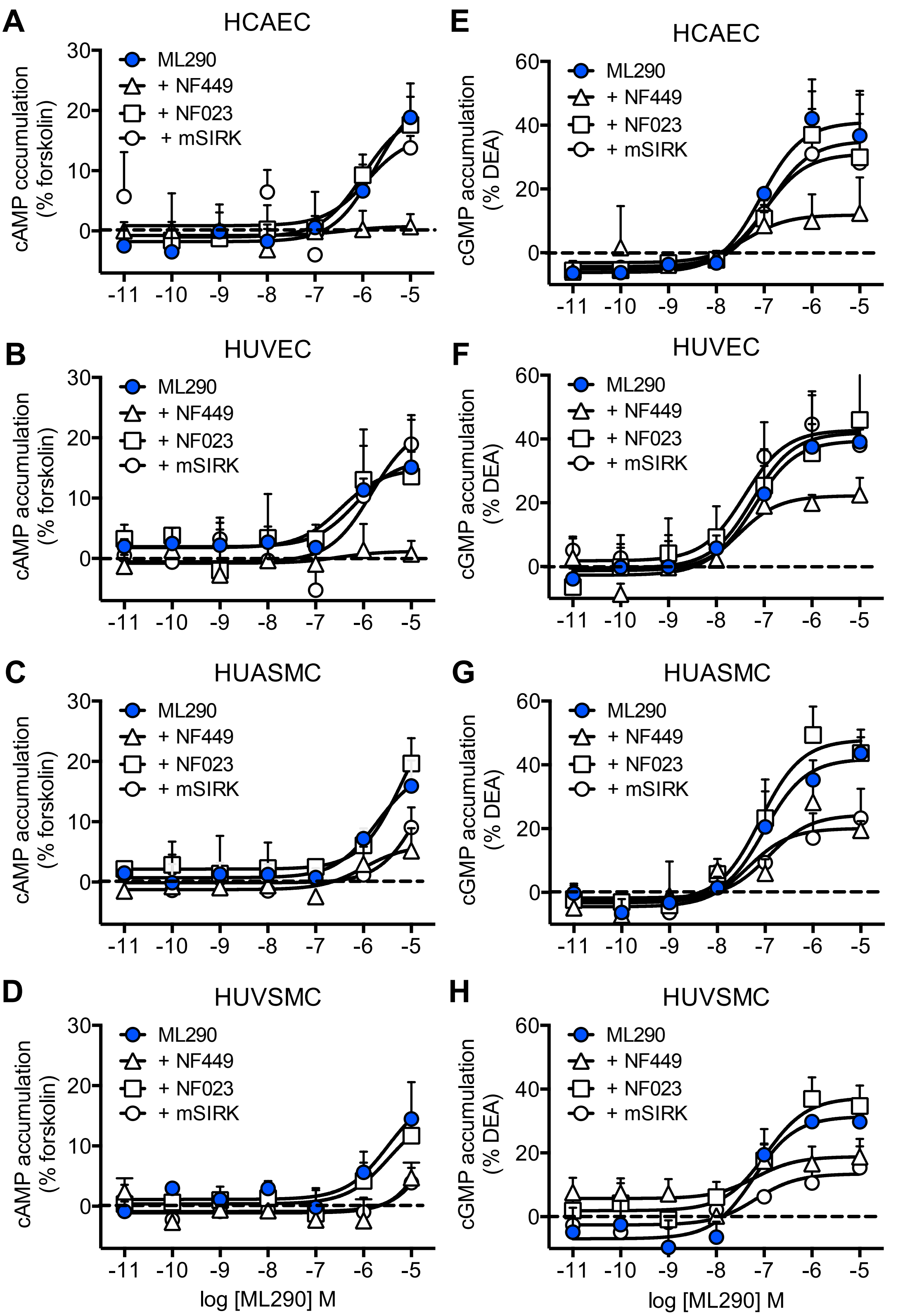

Figure S3 Role of G proteins and βγ subunits in ML290-mediated cAMP and cGMP accumulation in human primary vascular cells. *ML290 (30 min) increased cAMP accumulation in HCAECs (A; n=5), HUVECs (B; n=8), HUASMCs (C; n=8) and HUVSMCs (D; n=4). Treatment with the G*α_s *inhibitor NF449 (10*μ*M, 30min) in HCAECs (A; n=3) and HUVECs (B; n=4) abolished ML290-mediated cAMP accumulation (30 min) whereas in HUASMCs (C; n=4) and HUVSMCs (D; n=4) there was reduced maximal ML290-mediated*

cAMP accumulation. The $G\alpha_i/G\alpha_{OB}$ inhibitor NF023 (10μM, 30min) in HCAECs (A; n=4), HUVECs (B; n=4), HUASMCs (C; n=4) and HUVSMCs (D; n=4) had no effect on ML290-mediated cAMP accumulation (30 min). The $G\beta\gamma$ inhibitor mSIRK (5μM, 30min) in HCAECs (A; n=3) and HUVECs (B; n=3) had no effect on ML290-mediated cAMP accumulation (30 min) whereas in HUASMCs (C; n=3) or HUVSMCs (D; n=3) it reduced the maximum cAMP response (30 min).

ML290 (30 min) also increased cGMP accumulation in HCAECs (E; n=3), HUVECs (F; n=4), HUASMCs (G; n=3) and HUVSMCs (H; n=3). NF449 (10μM, 30min) in HCAECs (E; n=4); HUVECs (F; n=3), HUASMCs (G; n=6) and HUVSMCs (H; n=4) reduced the maximum ML290-mediated cGMP response (30 min). NF023 (10μM, 30min) in HCAECs (E; n=3), HUVECs (F; n=3), HUASMCs (G; n=4) and HUVSMCs (H; n=4) had no effect on ML290-mediated cGMP accumulation (30 min). mSIRK (5μM, 30min) in HCAECs (E; n=3) and HUVECs (F; n=3) had no effect on ML290-mediated cGMP accumulation (30 min) whereas in HUASMCs (G; n=5) and HUVSMCs (H; n=4) it reduced the maximum response. Data shown are mean ± SEM of 'n' independent experiments.

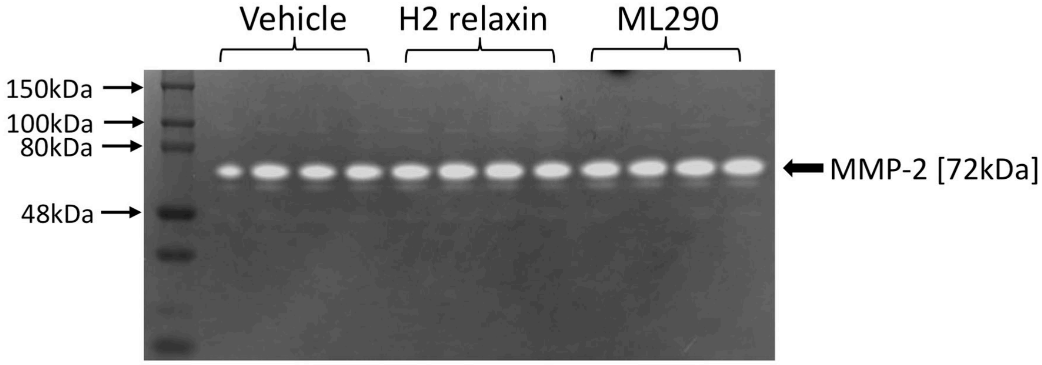

Figure S4 Full size zymograph of ML290 effects on MMP-2 expression (Figure 6) *ML290 (1μM) promoted MMP-2 activity to an equivalent extent to H2 relaxin (0.1μM) over 72 hours. Figure shows a representative zymograph of duplicate samples from two separate experiments*
